# Climate sensitivity is widely but unevenly spread across zoonotic diseases

**DOI:** 10.1073/pnas.2422851122

**Published:** 2025-12-09

**Authors:** Artur Trebski, Lewis Gourlay, Rory Gibb, Natalie Imirzian, David W. Redding

**Affiliations:** ^a^Science Department, Natural History Museum, London SW7 5BD, United Kingdom; ^b^Department of Genetics, Evolution and Environment, University College London, London WC1E 6BT, United Kingdom

**Keywords:** climate change, zoonotic diseases, global health, infection risk

## Abstract

Climate change is expected to exacerbate infectious diseases, yet the climate sensitivity of zoonotic diseases (driven by spillover from animal reservoirs) is understudied compared to vector-borne and water-borne infections. To address this gap, we conducted a scoping review and quantitative synthesis to identify relationships between climatic indicators (temperature, precipitation, humidity) and zoonotic disease risk metrics worldwide. We identified 218 studies from 65 countries describing 852 measures across 53 diseases, with most studies testing linear (n = 193) rather than nonlinear (n = 28) relationships. We found evidence of climate sensitivity across diverse zoonotic diseases (significant nonzero relationships in 69.1% of temperature effects, 63.5% of precipitation effects, and 53.6% of humidity effects), but with variation in direction and strength. Positive effects of temperature and rainfall on disease risk were more common than negative effects (46.5% vs. 22.6% and 37.8% vs. 25.7% of all records, respectively). These studies were predominantly located in areas expected to have substantial increases in annual mean temperature (>1.5 °C in 97% of studies) and rainfall (>25 mm in 53% of studies) by 2041 to 2070. Notably, the most consistent relationship was between temperature and vector-borne zoonoses (56% of positive effects, mean Hedges’ *g* = 0.36). Our analyses provide evidence that climate sensitivity is common across zoonoses, likely leading to substantial yet complex effects of climate change on zoonotic burden. We emphasize the need for future studies to utilize biologically relevant models, apply rigorous space-time controls, consider causal perspectives, and address taxonomic and geographic biases to allow robust consensus of climate–risk relationships to emerge.

## Significance

Understanding how climate change affects zoonotic diseases, those transmitted from animals to humans, is crucial for public health planning yet remains underexplored. Our global analysis of 218 studies covering 53 zoonotic diseases reveals widespread but uneven climate sensitivity among these diseases. Climatic factors, particularly temperature, are often linked to increased disease risk, especially for vector-borne diseases transmitted by arthropods. With many regions projected to experience significant warming, climate change may exacerbate zoonotic disease burden. However, few studies considered nonlinear effects, and the variation in responses both within and across diseases highlights complex dynamics demanding biologically informed research approaches. These findings underscore the urgent need to advance predictive frameworks to better anticipate and manage future disease risks in a changing climate.

The rapid increase in the emergence of zoonotic diseases and diseases of zoonotic origin, such as Zika, Ebola, and COVID-19, poses significant threats to global economies, public health, and social stability (1). The processes that drive pathogen spillover operate at the nexus of environmental change, socioeconomic structure, and public health (2). As global changes, including climate change, urbanization, and land-use transformation, reshape human–environment interfaces, the risk of novel patterns of zoonotic disease transmission rises (3–5). Among these drivers, climate change stands out as a particularly important yet still poorly understood factor (6), necessitating further research into how it influences the burden of zoonotic diseases across various systems.

Despite the zoonotic origins of most recent pandemics, previous research on climate change as a moderator of pathogen spillover has primarily focused on high-burden, vector-borne diseases (7–10). Moreover, biases in reporting efforts for neglected or emerging zoonoses—particularly in underresourced settings—add to the challenge of establishing baseline evidence (11, 12). Addressing these knowledge gaps is essential for developing realistic projections of how climate change may impact the global burden of zoonotic diseases (13). A better understanding of these dynamics is also crucial for developing global longitudinal monitoring programs, informing current research priorities, and helping identify potential trends in future zoonotic risk (3, 8, 14).

To extrapolate zoonotic disease risk into the future, it is vital to identify trends in the local-scale climate sensitivity of pathogens. Short-term variation in temperature, precipitation, and humidity alters disease transmission through multiple mechanisms (Table 1).

**Table 1.** Climatic drivers and their potential impacts on humans, reservoir hosts, and vectors, with illustrative case studies of disease outbreaks

	Example Impacts			Case Studies	
Driver	Humans	Reservoir Hosts	Vectors	Non-vectored	Vectored
**Low rainfall**	• Drought-induced food insecurity and physiological stress can reduce immunity • Drought can force reliance on poor, potentially contaminated water sources • People can store water in improvised containers • Dust and wind during droughts promote exposure to aerosolised pathogens	• Droughts push hosts such as rodents to invade human spaces in search for resources • Food scarcity can cause nutritional stress and lower immune resistance of hosts	• Water dependence can cause local extinction of population • Concentration effect: vectors may aggregate at remaining water sites • Vectors may invade alternative (e.g. human made) water reservoirs	**Q fever** • Incidence of Q fever was negatively associated with rainfall in Northern/Arctic region, with lower annual minimum precipitation corresponding to higher incidence, consistent with windborne dispersal of **Coxiella* burnetii* during dry, windy conditions and outbreaks occurring after drought (15, 16).	**Crimean-Congo Haemorrhagic Fever** • In southeast Iran, lower rainfall ~1 mo prior correlated with higher CCHF incidence, whereas accumulated rainfall ~5 mo prior increased incidence (17); see also ref. 18. Hyalomma ticks are most abundant in hot-desert (BWh) climates and overall abundances decline with precipitation (significant for *H. dromedarii* and *H. anatolicum*) (19). These short lags and arid-climate affinities are consistent with a vector-mediated pathway in which warm, dry periods heighten Hyalomma activity and human–livestock contact during animal handling (17–19).
**High rainfall**	• Intense rainfall or flooding can increase exposure to contaminated water • Infrastructure damage, crowding or displacement due to flooding can reduce sanitation • Reduced outdoor activity may lower exposure to insects	• Boosted plant growth and food availability can support larger host populations • Flooding can push hosts (e.g. rodents) out of their burrows, making them seek shelter in human areas or buildings	• Rain can create new water-based breeding sites for vectors • Increased plant growth can provide shelter and breeding sites for vectors • Extreme rainfall can flush away eggs or larvae from existing breeding habitats	**Leptospirosis** • In Brazil, a 20 mm weekly rainfall anomaly increased risk by 12% within few weeks, likely due to exposure to rodent-contaminated water in poorly drained areas (20). • In Fiji, severe flooding following cyclones was linked to outbreaks, with water contaminated by both rodent and livestock urine driving transmission (21).	**Yellow Fever** • In the Americas, rainy-season conditions can elevate risk by expanding tree-hole breeding and survival of sylvatic vectors (*Haemagogus/Sabethes*); at continental and seasonal scales, rainfall amplitude and intensity are among the most informative predictors of human reports (22–24). • An exception is the 2017 to 2018 Brazil epidemic, which coincided with severe drought which likely increased host-vector contact and promoted aggregation at reduced suitable habitat (25).
**Heat**	• Reduced immune defences due to heat stress • Heat can induce behavioural changes, including opening windows or evening outdoor activity increasing evening vector exposure • Increased use of water reservoirs can increase exposure to vectors	• Milder winters in cooler regions can reduce winter mortality of host populations • Sustained increases in temperature enable hosts to expand their geographical ranges • Higher temperature increases food availability by promoting plant growth	• Survival reduction in heat beyond thermal optimum • Ectotherms experience accelerated metabolism and reproduction rates in higher temperatures while below thermal optimum • Pathogens within vectors can develop faster in higher temperatures, turning vectors infectious faster	**Puumala virus** • Across West Europe and Scandinavia, PUUV risk aligns with colder winters and milder summers: warmer winters and summers reduce bank-vole seroprevalence, and high-risk regions have colder winters, consistent with greater viral environmental persistence (26–28). • By contrast, in Northern/Arctic settings, milder winters coincide with higher incidence, likely via reduced snow cover and increased rodent-human contact (16).	**West Nile Virus** • In the US, higher annual and winter temperatures were associated with increased WNV risk, likely due to enhanced mosquito survival and transmission efficiency (29, 30). • In southern Russia, WNV outbreaks were linked to warm May–July temperatures (>21 °C), which accelerated virus replication and mosquito activity (30, 31).
**Humidity**	• Wet bulb effect results in increased heat stress under higher humidity, rising susceptibility to infections • Wearing lighter or looser clothes due to higher humidity can increase exposure to biting vectors • Low relative humidity increases dust generation, raising inhalation exposure to aerosolised pathogens	• Higher humidity supports denser vegetation, increasing food provisioning and shelter to hosts • High humidity can impair thermoregulation of hosts and induce heat stress, potentially increasing susceptibility to infections	• High ambient humidity improves vector survival, for example preventing ticks from desiccation • Increased humidity allows higher egg and larvae survival • Vectors exhibit increased activity and biting rates under higher humidity	**Brucellosis** • In Iran, monthly incidence correlated positively with humidity at lags up to ~5 mo (32). In China, analyses detected lagged effects of humidity on incidence (33). However, other studies found null or negative associations, indicating heterogeneous impacts (34–36). • Humidity can prolong *Brucella* survival in moist environments whereas dry, windy conditions may favor aerosol dispersion, pathways that could produce opposing patterns across sites.	**Japanese encephalitis** • Across multiple settings, higher relative humidity significantly increased the risk and intensity of JE outbreaks. In Chongqing, humidity increased both outbreak risk and intensity, and mosquito density in humid livestock sheds further elevated risk (37). Positive humidity–JE associations were also identified in Linyi and Jinan cities (China) and in Taiwan (38–40). • Mechanistically, high humidity can enhance survival, reproduction, and feeding activity of *Culex* mosquitoes, which breed largely in humid livestock sheds and agricultural areas.

**Fig. 1.**
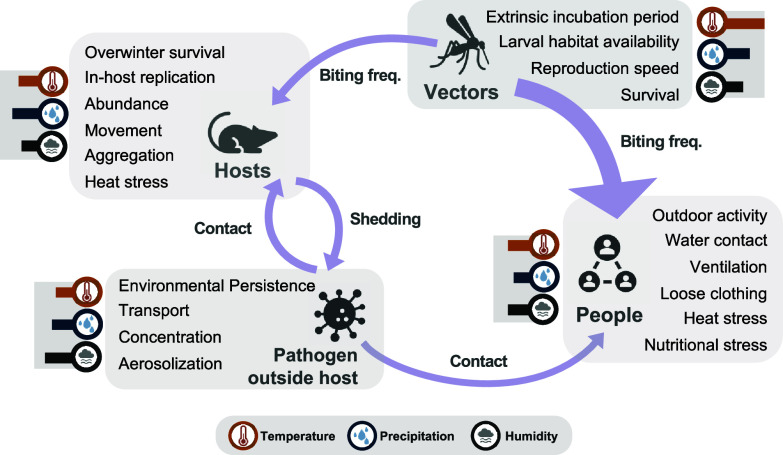
Conceptual schematic of zoonotic transmission with and without an arthropod vector. Gray boxes list examples of climate-sensitive intrinsic factors. Arrows indicate key transmission processes that may be climate-sensitive; arrow width is illustrative of the relative evidence of impact on disease risk. Small bar charts show hypothesized weightings of temperature, precipitation, and humidity for each component. These pathways and weightings are indicative, not exhaustive, and will vary by pathogen, place, and season.

Temperature changes can impact life-history rates in vectored systems, including biting and oviposition rates, development speed, survival, and pathogen extrinsic incubation period, which yields a unimodal thermal performance where risk rises toward a system-specific thermal optimum and declines beyond it (30, 41). In nonvectored systems, temperature also influences pathogen replication/survival outside the host, as well as reservoir phenology, movement, body condition, and shedding, and modifies human exposure behaviors (e.g., time outdoors, ventilation) (41–44). While precipitation plays a key role in creating larval habitats and moist refugia, flooding mobilizes contamination and increases water contact, and drought concentrates hosts and humans at scarce water sources, often driving increased household water storage and resulting in abundant artificial vector breeding sites (20, 21, 45–47). Humidity constrains tick and mosquito survival/activity, mediates environmental persistence vs. aerosolization trade-offs for environmental pathogens (high humidity favors persistence; low humidity favors airborne transport/dust), and intensifies heat stress (33, 48, 49). While all three climate factors influence transmission, the evidential strength is uneven (Fig. 1). Mechanistically, temperature effects on ectotherm vectors are among the most consistently demonstrated; the mechanisms and outcomes linked to precipitation and humidity are less consistent and strongly context-dependent (49).

Examples of empirically studied zoonotic climate sensitivity encompass a range of transmission modes and disease types. Heavy rainfall and flooding mobilize *Leptospira* and increase water contact, increasing leptospirosis risk (20, 21). Dry, windy conditions following low rainfall favor aerosolization of *Coxiella* and Q fever outbreaks (15, 16). In arid settings dominated by *Hyalomma* ticks, lower rainfall increases Crimean-Congo Hemorrhagic Fever risk (17–19). In turn, warmer spring–summer conditions accelerate within-vector dynamics and elevate West Nile virus risk (29–31). Moreover, higher relative humidity in moist, rain-fed agroecosystems expands *Culex* habitat and activity, raising Japanese encephalitis risk (37–40). Finally, in Europe and Scandinavia, Puumala virus risk tracks winter conditions (colder winters favor persistence), with contrasting increases under milder winters in Northern/Arctic settings (16, 26–28). Further examples of zoonotic climate sensitivity mechanisms and case studies are summarized in Table 1.

Given the broad evidence of climate sensitivity in zoonotic diseases, transmission risk is likely to be impacted by ongoing climate change. Long-term climate changes may alter large-scale movements of key hosts and vectors, modify land-use patterns, and change basic physiological responses, with consequences for disease susceptibility and transmission (3, 44, 50–52). This may both exacerbate and suppress different elements of the spillover pathway, creating potentially complex effects that vary across systems (53). Improving our knowledge of the response of reservoir hosts to climate variability will help disentangle the potential impacts of climate change on disease risk. While many mosquito-vectored diseases of humans are well studied in relation to climate variability (30), the climate sensitivity of zoonoses more broadly is poorly defined, despite representing an essential dimension of public health risk.

Recent reviews and analyses have addressed some key questions in this area. Many studies have identified increasing disease risk stemming from climate change (5, 41, 53, 54), yet they reveal mixed findings on the consistency of these responses, with some showing a high degree of context-dependent effects. Notably, a gap remains in research specifically addressing the sensitivity of diverse zoonotic disease systems to different aspects of climate. By synthesizing globally distributed empirical studies on various climate metrics and disease risk measures, this study aims to evaluate how climate sensitivities apply across zoonoses. Zoonotic diseases may exhibit distinct responses to climate drivers compared to nonzoonotic infections due to their complex transmission ecology. Unlike directly transmitted human pathogens, zoonoses involve animal reservoir hosts, and often arthropod vectors, each with unique physiological tolerances, habitat requirements, and behavioral responses to climatic conditions (Table 1). In addition, we hypothesize that vector-borne zoonoses are more likely to exhibit stronger or more consistent responses to climate drivers, especially temperature, due to the well-defined thermal sensitivity of ectotherm vectors and pathogen replication (30).

Here, we conduct a comprehensive analysis of primary studies that evaluate the empirical relationships between climatic parameters (i.e., temperature, precipitation, and humidity) and measures of zoonotic risk (i.e., abundance, seroprevalence, number of cases, and incidence) to assess the state and consistency of evidence for climate sensitivity across zoonotic disease systems. Specifically, our study aims to: 1) identify which regions and zoonotic diseases are over- or underrepresented in climate sensitivity research; 2) determine whether any consistent patterns emerge in climate sensitivity across disease types, regions, types of vectors, and hosts; 3) evaluate whether the methods and metrics used in source studies are appropriate to detect such trends; and 4) assess whether climate sensitivity differs by transmission pathways.

## Results

### Literature Search Results.

**Fig. 2.**
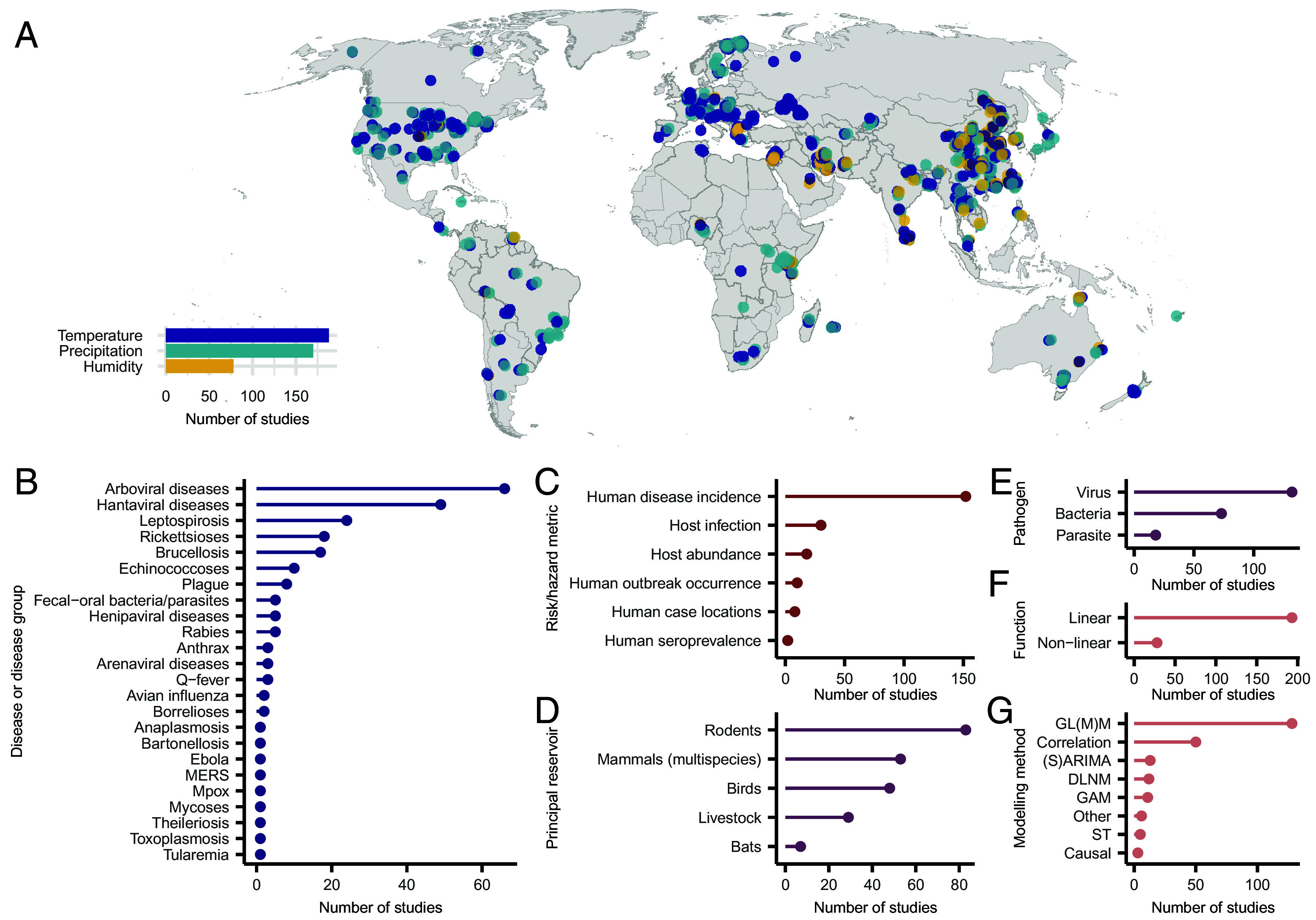
Overview of compiled studies examining the effects of climatic factors on zoonotic disease transmission. (*A*) Map showing the full database of extracted climate effects (n = 852 effects, 218 studies), summarized geographically, with point color representing the climatic driver, and location representing the specified lat-lon or nearest named locality of the study. Studies without locality information were geolocated to the country centroid. The inset barplot in (*A*) shows the number of studies reporting effects of temperature, precipitation, or humidity on zoonotic diseases. Subplots show the database broken down by key variables: (*B*) disease or broad disease grouping; (*C*) the risk or hazard metric being tested in the study; (*D*) the principal reservoir host(s) of the study’s focal disease; (*E*) the broad pathogen type; (*F*) whether the study reported linear or nonlinear inferred effects; and (*G*) the broad type of modeling method used. Method abbreviations used in (*G*): GL(M)M, generalized linear (mixed effects) model; (S)ARIMA, (seasonal) autoregressive integrated moving average model; DLNM, distributed-lag nonlinear model; GAM, generalized additive model; ST, spatiotemporal statistical model; Causal, an explicitly causal inference-based model; Other, descriptive or basic frequentist statistics (e.g., chi-square test).

The literature search, guided by detailed search criteria (*SI Appendix*, Table S1), yielded 14,470 article titles, from which 218 empirical studies (1.5%) met the inclusion criteria after a four-step screening process (*SI Appendix*, Fig. S1). These studies encompassed 53 zoonotic diseases (*SI Appendix*, Table S2) across 65 countries, providing 852 statistics quantifying the relationship between climate variables and measures of zoonotic disease risk. Temperature and precipitation were the most frequently examined variables, representing 49% (n = 414) and 38% (n = 326) of the extracted relationships, respectively, while humidity accounted for 13% (n = 112). The studies had close to global coverage, with areas of high sampling in South and East Asia and Europe, and more limited sampling in Central and North Asia and Eastern Africa (Fig. 2*A*). Arboviruses were the most frequently studied group of pathogens (28.8%, n = 66), followed by Hantaviruses (21.4%, n = 49) and *Leptospira* spp. (10.5%, n = 24) (Fig. 2*B*).

### Studies Often Report Increased Zoonotic Risk under Warmer and Wetter Conditions.

Most studies reported significant associations between climatic factors and zoonotic disease risk, with 68.7% of the total 852 relationships being significant at α = 0.05, and 42% remaining significant at α = 0.01. Reported positive associations, indicating increased disease risk with warmer or wetter conditions, were 1.7 times more common than negative relationships (χ^2^ = 32.94, df = 1, 95% CI = 32.24 – 33.65, *P* < 0.001, from 1,000 bootstraps of 80% of the data). The proportions observed within the overall dataset remained robust after excluding most major regions, disease groups, pathogen types, statistical methods, and hosts from the calculations (*SI Appendix*, Table S3). The predominance of reported positive relationships was particularly pronounced among vector-borne diseases (194 positive vs. 82 negative; χ^2^ = 39.46, df = 1, *P* < 0.001), whereas nonvectored diseases showed more balanced proportions of positive and negative associations (157 positive vs. 120 negative; χ^2^ = 4.14, df = 1, *P* = 0.185).

**Fig. 3.**
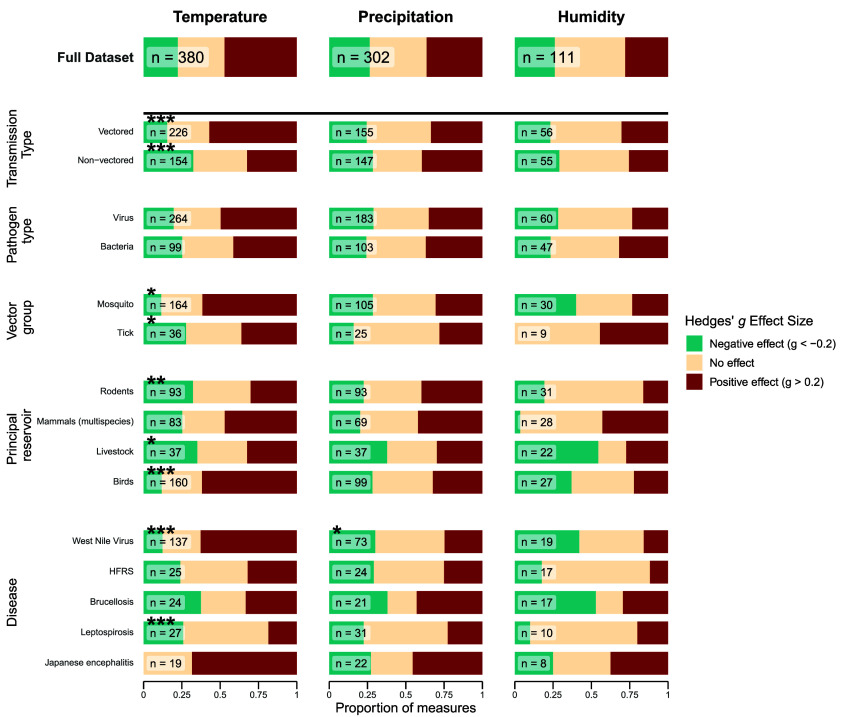
Direction of climate effects across key disease variables. Each column represents the climate driver studied, with the x-axis indicating the proportion of study measures falling into effect categories from a negative effect (blue) to no effect (cream) to a positive effect (red). The rows separate the data into the key variables identified in Fig. 2: the main transmission mode (vectored via a biting vector from reservoir host to human, or nonvectored, with no involvement of a vector between reservoir host and human); the broad pathogen type; for the vector-borne diseases, the broad animal group of the vector; and the principal reservoir host(s) of the study’s focal disease. Groupings were included if the category had greater than 15 effect size measures. The number of measures included in each grouping is indicated next to the bars. Distributions that were significantly different from the full dataset based on Anderson–Darling tests are marked with asterisks above the sample size (*P < 0.05; **P < 0.01).

**Fig. 4.**
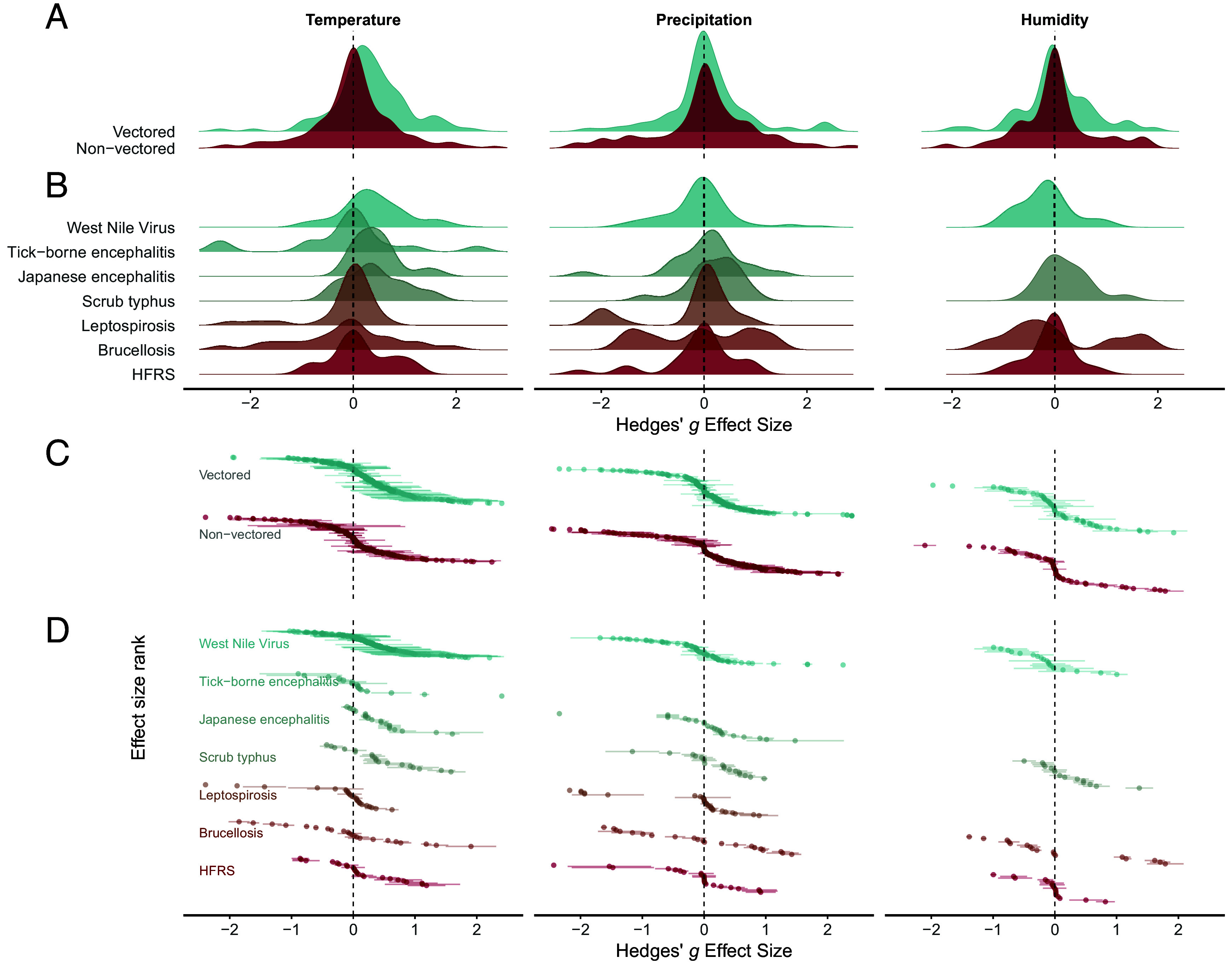
Comparison of effect size distribution across diseases and transmission modes. (*A*) Ridgeline density plots showing the distribution of Hedges’ g values calculated from studies measuring disease risk in response to three climate variables: temperature, precipitation, and humidity (three columns, left-to-right). The bluish densities represent vectored diseases, and the reddish densities represent nonvectored diseases. (*B*) Density plots as in (*A*) but divided by disease; shown are West Nile virus, Tick-borne encephalitis, Japanese encephalitis, Scrub typhus, Leptospirosis, Brucellosis, and HFRS (hemorrhagic fever with renal syndrome), for which at least fifteen effect-size estimates were available for each climate variable. (*C*) Snake-style scatterplots displaying the individual effect sizes contributing to vectored and nonvectored distributions in (*A*); each point represents one effect size, ordered on the *y*-axis from lowest to highest values. Lines show 95% CI where the data permitted such calculations. (*D*) Scatterplots as in (*C*) but divided by disease, corresponding to the set shown in (*B*). Extreme values (Hedges’ g values less than −2.5 or greater than 2.5) were excluded from the plot.

While climatic variables in general significantly influenced zoonotic disease risk across diverse study systems, the direction and magnitude of standardized effect sizes varied notably among climatic factors reported and between pathogen groups (Figs. 3 and 4). Across these finer scale categories, temperature consistently emerged as the most influential climatic variable, with 70% of reported effect sizes indicating significant climate sensitivity (Hedges’ g ≠ 0). Positive associations with rising temperatures dominated these findings (Fig. 3), particularly for pathogens such as West Nile Virus, Scrub typhus, and Japanese Encephalitis (Figs. 3 and 4 *B* and *D*).

By contrast, precipitation and humidity responses lacked a clear trend, with similar positive and negative proportions and higher “no-effect” proportions (humidity 46.4%, precipitation 36.5% vs. temperature 30.9%; Fig. 3). Precipitation displayed some notable positive associations for certain diseases, particularly Japanese encephalitis, Scrub typhus, and Leptospirosis (Fig. 4 *B* and *D*).

### Vector-Borne Zoonoses Show Stronger and More Consistent Climate Sensitivity Compared to Other Transmission Modes.

Vector-borne zoonotic diseases exhibited the most substantial evidence of climate sensitivity, particularly regarding temperature effects. Mosquito-vectored and tick-borne diseases, as well as vector-borne diseases overall, and systems with birds, rodents, or livestock as primary reservoirs consistently differed from the overall temperature–effect distribution (Anderson–Darling tests; Fig. 3 and *SI Appendix*, Table S4), with particularly strong signals for West Nile Virus. Notably, vector-borne zoonoses were reported to have significantly more positive temperature associations than non-vector-borne diseases (Kolmogorov–Smirnov test, *P* < 0.0001; Figs. 3 and 4 *A* and *C* and *SI Appendix*, Table S5). While the trends for nonvectored diseases were less pronounced, 32% of temperature effect sizes for these diseases were positive (compared to 56% for vector-borne diseases), indicating that temperature increases may still elevate the risk for many nonvectored zoonotic diseases.

Unlike temperature, no clear pattern emerged for responses to precipitation or humidity. The overall effect sizes for these climate variables generally centered around zero, without significant differences between the distributions among vectored and nonvectored subsets (Kolmogorov–Smirnov test; precipitation: *P* = 0.377; humidity: *P* = 0.501; Fig. 4 *A* and *C* and *SI Appendix*, Table S5). However, West Nile Virus demonstrated distinct sensitivity to precipitation, diverging significantly from responses of other diseases to this climate factor (Anderson–Darling tests; Figs. 3 and 4 *B* and *D* and *SI Appendix*, Table S6). Although the distribution of precipitation effect sizes had visually larger tails for nonvectored diseases compared to vectored diseases (Fig. 4 *A* and *B*), Anderson–Darling tests revealed no statistically significant differences between these groups or any other subset of responses to precipitation analyzed (*SI Appendix*, Table S7).

### Projected Climate Shifts at Study Locations May Favor Increases in Zoonotic Transmission Risk.

We identified that many study sites associated with climate-sensitive diseases (those with Hedges’ g > 0.2 or < −0.2) are in regions where future climatologies consistently predict substantial temperature changes (BIO1, mean annual air temperature) or precipitation (BIO12, annual precipitation). Climate projections, derived from the CHELSA V2.1 CMIP6 dataset, were assessed across three Shared Socioeconomic Pathways (SSP1-RCP2.6, SSP3-RCP7.0, SSP5-RCP8.5) and five Global Climate Models for the period 2041 to 2070 relative to baseline conditions (1981 to 2010). Climate shifts were categorized according to thresholds for temperature (1 °C, 1.5 °C, and 2 °C) and precipitation changes (±25 mm, ±50 mm, and ±100 mm), identifying the most consistent predictions at each location and threshold.

**Fig. 5.**
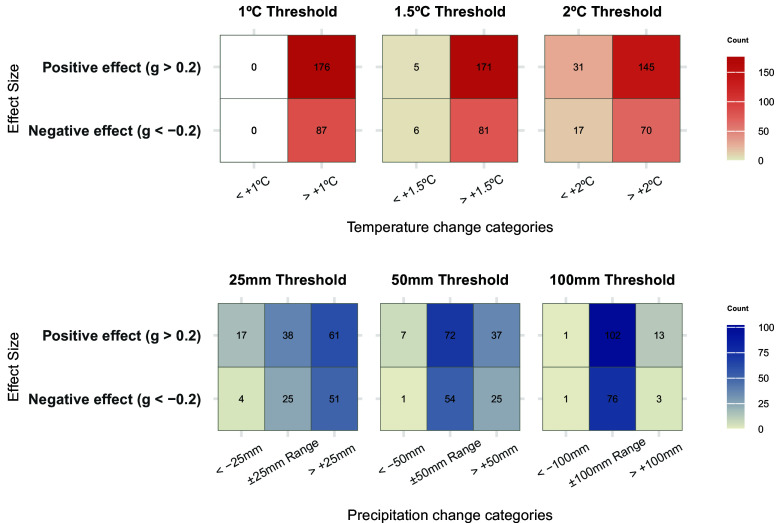
Contingency tables showing the association between disease climate sensitivity effect sizes (Hedges’ *g*) and predicted changes in temperature and precipitation across study locations, based on future climate projections from the CHELSA V2.1 CMIP6 dataset. The *Top* row represents temperature thresholds of 1 °C, 1.5 °C, and 2 °C, while the *Bottom* row represents precipitation thresholds of ±25 mm, ±50 mm, and ±100 mm. Positive (Hedges’ g > 0.2) and negative (Hedges’ g < −0.2) climate sensitivity effect sizes are shown in relation to predicted climate change categories. The numbers in each tile represent the count of locations where the most frequent (modal) category of climate change, determined from 15 projections (comprising five Global Climate Models and three Shared Socioeconomic Pathways), was associated with the respective sensitivity effect size.

At sites associated with positive temperature–risk effects, all locations exceed +1 °C, 97% exceed +1.5 °C, and 82% exceed +2 °C of projected warming (Fig. 5). Sites with negative temperature–risk effects show a similar distribution of projected warming (100% > +1 °C; 93% > +1.5 °C; 80% > +2 °C). Formal tests using contingency tables with 1,000 bootstrap replicates found no significant association between effect-size sign and warming magnitude (*SI Appendix,* Table S8; Fisher’s exact at +1.5 °C: *P* = 0.30; χ^2^ at +2 °C: *P* = 0.56).

Projected changes in precipitation were more heterogeneous across sites than temperature and showed no association between the sign of the precipitation-risk effect and the magnitude of projected precipitation change (*SI Appendix*, Table S8). Among sites with positive precipitation-disease risk effects, 53% are projected to see increases in annual precipitation exceeding +25 mm, 32% exceed + 50 mm, and 11% exceed +100 mm (Fig. 5). Substantial decreases in precipitation were rarely projected; among sites with negative precipitation-risk effects only 5% exhibit reductions greater than 25 mm, and just 1% exceed 50 mm or 100 mm. Contingency-table tests at ±25, ±50, and ±100 mm were nonsignificant (mean *P* = 0.13, 0.24, 0.19, respectively; *SI Appendix,* Table S8), indicating no detectable association between effect-size sign and projected precipitation change.

### Data Biases and Methodological Inconsistencies Limit a Clear Synthesis of Climate Sensitivity in Zoonoses.

Several methodological limitations were identified within the collated dataset and source studies. While Hedges’ *g* could be calculated for 812 of the measures extracted (95%), the respective sample sizes had to be inferred or calculated based on the study design for 60% of the records. This often relied on reconstructed variance from reported SE/SD/CI or test statistics, introducing approximation-related uncertainty.

The dataset analyzed contains considerable diversity in the number of data points per disease and the statistical methods employed across studies. Certain diseases, such as West Nile virus (n = 29 studies), hemorrhagic fever with renal syndrome (n = 28 studies), and leptospirosis (n = 23 studies), were relatively well-represented. In contrast, eight diseases (anaplasmosis, bartonellosis, Ebola, MERS, mpox, theileriosis, and toxoplasmosis) were represented by only one study each. Even among well-represented diseases, the effect sizes were highly heterogeneous in both magnitude and direction (regardless of transmission type), limiting the identification of clear patterns of climate sensitivity (Fig. 4 *B* and *D*).

In terms of methodology, the collated dataset represented 103 specific statistical methods used in the source studies, many of which lacked consistent and detailed reporting of the methods used. The use of nonlinear models was relatively rare, with only 13% of studies investigating nonlinear relationships between climatic variables and measures of zoonotic disease risk (Fig. 2*F*).

Overall, most studies relied on relatively simple statistical models (Fig. 2*G*): 128 studies (56.1%) used generalized linear or mixed-effects models (GL(M)Ms), followed by 50 studies (21.9%) using correlation approaches. A smaller subset employed seasonal autoregressive models ((S)ARIMA; 13 studies, 5.7%), distributed lag nonlinear models (DLNMs; 12 studies, 5.2%), or generalized additive models (GAMs; 11 studies, 4.8%). Spatiotemporal models were used in 5 studies (2.2%), while explicitly causal frameworks were used in just three studies (1.3%).

Within the broad GL(M)M category, which was the most used framework, around half of the studies (46%) employed standard generalized linear models, typically testing only main effects. Linear models accounted for 20%, 14.8% used generalized estimating equations, while only 8.5% used mixed-effects models capable of handling hierarchical or nested data structures. The remaining studies used time-series regression models (4.1%), zero-inflated regressions (3.3%), or Bayesian regression approaches (2%). The heavy reliance on generalized linear models, linear regression and correlation methods, and time-series models may oversimplify the complexities of climate–disease relationships.

There was some degree of publication bias inferred from funnel plots showing SE against respective effect sizes (*SI Appendix*, Fig. S3). Egger’s regression tests for funnel plot asymmetry showed significant asymmetry in the overall dataset (z = 4.8, *P* < 0.001), temperature data (z = 3.07, *P* = 0.002), and humidity data (z = 3.83, *P* < 0.001), but none within precipitation data (*SI Appendix*, Fig. S3 and Table S9). Pearson’s product-moment correlation analysis showed no significant correlation between OpenAlex 2-y mean citedness and Hedges’ g or reported *P*-values, even after removing outliers among Hedges’ g and *P*-values (*SI Appendix*, Fig. S2). Furthermore, *P*-values extracted from the studies were examined to check for potential evidence of “p-hacking”. Visual inspection of the distribution of *P*-values surrounding *P* = 0.05 found limited evidence of p-hacking, with a small spike in the number of studies with *P*-values just under *P* = 0.05 (*SI Appendix*, Fig. S4).

## Discussion

Our study provides evidence that climate sensitivity is widely spread across a diverse range of zoonotic diseases, suggesting that climate change is likely to impact patterns of future risk. However, significant biases and methodological gaps in current research hinder our ability to predict specific mechanisms, locations, and magnitudes of these impacts. By identifying the variability and limitations in our knowledge, we highlight the urgent need for standardized and transdisciplinary approaches to better predict how, where, and through which mechanisms climate change will affect the global burden of zoonotic diseases.

### Temperature Effects Are Strongest and Most Consistent in Vector-Borne Zoonoses.

The clearest result from our analysis is the positive temperature–risk association for zoonotic diseases involving arthropod vectors in the transmission cycle (Figs. 3 and 4), consistent with prior evidence documenting links between climate change and the spread of mosquito- and tick-borne diseases such as Japanese encephalitis, Lyme, and West Nile virus into novel regions (13). This finding also aligns with conceptual syntheses that suggest vector-borne diseases are among the most climate-sensitive groups, due to multiple temperature-dependent traits within the transmission cycle (49).

Moreover, we found that nearly all study sites with positive temperature effect sizes are in regions projected to experience temperature increases exceeding 1.5 °C, with many also surpassing the 2 °C threshold (Fig. 4). Although this overlap could translate into heightened transmission risk, the effect will depend on where each site sits on the unimodal temperature–risk curve: locations still below the thermal optimum may indeed see amplification of disease risk, whereas sites nearing or exceeding that optimum could experience plateauing or even declining risk (30, 55, 56). Importantly, disease risk responses to climate can appear minimal or undetectable near thresholds and on plateaus, as demonstrated in the West Nile virus system, where sensitivity to changing temperature concentrates within a narrow transitional temperature window (21 to 22.7 °C), with little change in transmission risk outside this temperature range (57).

Mechanistically, warming can increase vectorial capacity by accelerating rates of multiple processes, including a higher frequency of biting and egg-laying, faster pathogen replication, and a shorter extrinsic incubation period (EIP). In some systems, this may also lead to improved immature development and adult survival, up to a system-specific thermal optimum (30, 58). Taken together, these trait sensitivities produce the unimodal temperature–risk curve described above (30, 55, 56, 58, 59). Moreover, temperature variability (daily/seasonal) can shift apparent optima, so temporal means alone may further mischaracterize sensitivity, especially where variance is high. These patterns align with the thermal biology literature on mosquito-borne infections and help explain why temperature signals in our synthesis are strongest for vectored systems, although we note that there are still clade and climate-specific responses (e.g., see CCHF in Table 1).

By contrast, nonvectored systems are characterized by indirect, lagged, and sometimes opposing pathways; consequently, temperature responses along the transmission cycle can be more heterogeneous compared to vectored systems. In an environmental stage, the persistence of microorganisms often exhibits narrow thermal windows: high heat and desiccation can reduce survival, whereas warmth with moisture can increase persistence and growth—e.g., *Leptospira* in flood-prone or waterlogged settings, yielding thresholds and plateaus rather than monotonic increases (6, 20, 21, 43). In the host stage, temperature can alter overwinter survival and recruitment (e.g., bank voles and HFRS dynamics) and produce system-specific lags (26–28, 60). In livestock and poultry, heat stress, birth pulses, and aggregation at shade or water sources alter contact networks and shedding, while hot and dry periods can increase the spread of pathogens via aerosols (43, 44, 61, 62). In bird reservoirs, warming can also shift migration timing, aggregation, and weaken or strengthen immune function at wildlife–livestock–human interfaces, although these signals are frequently modulated by moisture and land use (54, 63–65). Together, these host and environment-mediated pathways help explain why temperature effects for nonvectored systems are more heterogeneous, as seen in our comparison of vectored and nonvectored effect-size distributions (Fig. 4 and *SI Appendix*, Table S5).

### Precipitation and Humidity Produce Context-Dependent, Bidirectional Impacts.

In contrast to temperature effects, we did not find consistent evidence that precipitation and humidity exert strong unidirectional effects on zoonotic disease risk. Rather than indicating a common direction of effect, precipitation shows a wider, bidirectional spread of effect sizes, with more of the large positive and negative values than temperature and many near-zero associations (Fig. 4). This pattern is mechanistically coherent with multiple, sometimes opposing pathways through which moisture acts on physical resource dynamics, aerosol transmission, habitat integrity, and human exposure.

For example, for water-borne zoonoses such as leptospirosis, we might expect a unidirectional relationship, as heavy rainfall and flooding can coincide with increased transmission due to enhanced exposure to contaminated water sources. Rainfall and flooding can mobilize *Leptospira* from rodent or livestock urine into drains, sewers, and surface waters, promoting contact during routine activities and postflood clean-up, especially in areas with poor drainage and sanitation.

For rodent-borne zoonoses that are transmitted to humans via aerosolized excreta, such as Hantavirus systems (e.g., HFRS, HPS), moisture can act through multiple pathways: indirect effects on rodent demography (food pulses, overwinter survival, movement), and direct impact on aerosol generation, virus persistence, and human–rodent contact that depend on rainfall intensity, humidity, and timing. The net effect can be strongly seasonal and lagged, producing both positive and negative associations across settings (Fig. 4).

There are various complex and lagged relationships in specific systems. For instance, HPS incidence rises with rainfall at 2 to 6 mo lags, consistent with rainfall-driven resource pulses and subsequent human exposure (66). In Northern Europe, above-average November rainfall increases the subsequent spring seroprevalence of Puumala virus, independent of rodent abundance, possibly by concentrating rodents into scarce, dry refugia, and increasing social contact, or by imposing physiological stress that may heighten susceptibility, thereby accelerating winter transmission (67).

Conversely, higher relative humidity and increased rainfall are associated with fewer HFRS cases after a lag, possibly due to lower survival and stability of the virus outside the host, and decreased rodent and human activity (68). However, at larger spatial scales, province-level analyses and European studies have found mixed, context-dependent precipitation signals, with only weak, study-specific evidence for humidity. Heterogeneity in lag and seasonal windows is likely driving the mixed results (69, 70). These opposing pathways are consistent with the broad, bidirectional spread precipitation distributions we estimate for rodent-borne diseases with aerosol transmission (Fig. 4), with seasonal/lag structure further widening the spread.

For vector-borne zoonoses, we show less consensus on the effects of precipitation than for temperature. Using the example of Japanese encephalitis (JE), most precipitation associations are neutral with a skew toward positive Hedges’ *g*, among nonneutral effects. Positives are more common with 1 to 2 mo lags, consistent with monsoon rains and paddy irrigation expanding larval habitat, increasing adult longevity via higher humidity, and allowing time for within-vector incubation and amplification in pigs (72–74). However, this is not a linear relationship, as very intense or persistent rainfall and short, heavy bursts can flush larvae or damage habitats, yielding inverse associations or downturns at high rainfall (74–76). Regional heterogeneity and model structure also matter: pig seroconversion data show both positive and negative precipitation correlations depending on region and lag (76), and analyses in Chongqing and Guizhou found no relationship with precipitation once temperature, humidity, socioenvironmental covariates, and vaccination history were accounted for (37, 77). Collectively, these patterns support a bidirectional, context-dependent precipitation effect for JE: moderate, lagged rainfall and irrigation often elevate risk via habitat expansion and humidity, whereas extremes and high-frequency downpours can suppress risk through flushing, with land use, pig density, and vaccination shaping the observed signal.

Such contrasting dynamics and heterogeneous responses to climatic factors help explain the absence of consistent precipitation and humidity effects on overall disease risk within our dataset. They also highlight some reasons why water-borne zoonoses, such as leptospirosis, tend to show more uniformly positive responses to moisture, while airborne diseases such as HFRS/HPS and vectored diseases, including Japanese encephalitis, exhibit mixed, and context-dependent outcomes under similar conditions (Fig. 4 *B* and *D*). Without more consistent and representative data, however, the disease risk relationship with precipitation and humidity will likely remain uncertain, though we note that most of the sites that found positive precipitation effects are projected to experience substantial rainfall increases, suggesting that for these regions and these diseases, at least, we expect to see elevated risks.

### Methodological and Data Limitations Complicate Inference.

There are numerous biases and limitations across the zoonotic disease literature that hinder our ability to generalize findings. While the majority of source studies report statistically significant associations between climate factors and zoonotic risk, this high proportion is likely inflated by publication bias, as null results are less frequently published (see the *Data Biases* section and *SI Appendix*, Fig. S3). The analyzed studies predominantly focus on rodent-borne diseases, with bat-associated diseases notably underrepresented, despite their key role in recent high-impact spillover events, such as Ebola and COVID-19, and the general uptick in epidemiological studies examining bat-borne diseases since 2003 (11). Indeed, examining the widely different ecologies of bats and rodents could help understand the role of mobility in escaping climate effects; however, given the paucity of data for bats (n = 16), a meaningful comparison was not possible at this stage. Overall, we identified studies on only 53 diseases, which represents just ~6% of the (at least) 816 known human zoonotic pathogens (78). This taxonomic and geographic bias highlights the need for a more comprehensive and globally representative approach to studying the climate sensitivity of zoonotic diseases.

Methodological limitations further obstruct our understanding. Over half of the studies (60%) did not report a precise sample size used to calculate reported statistics, making it difficult to assess the reliability of their results. Although we identified over 50 distinct statistical methods reported, most studies either did not use or did not clearly state that they used the methods that can control for ubiquitous biases often seen in epidemiological data, such as spatial and temporal autocorrelation, and detection and reporting biases. Moreover, many studies contributed multiple effect sizes (e.g., for temperature, precipitation, and humidity across various subregions), which may not be fully independent, potentially inflating significance and underestimating uncertainty. Therefore, undertaking a formal meta-analysis, which is ideally intended to inform policy actions, is challenging and unlikely to lead to robust findings at this stage.

Additionally, many models lacked biological justification. Both theoretical (30, 41) and empirical evidence (45, 46, 55, 56, 79, 80) suggest that relationships between climate and pathogen transmission are often complex and nonlinear, depending on the thermal biology of the vectors and pathogens involved. Yet only 13% of the studies we found investigated these nonlinear effects. As mentioned previously, many pathogens and vectors, particularly arthropods, have unimodal (“hump-shaped”) thermal performance curves, in which factors crucial for transmission (e.g., vector survival, biting frequency, pathogen replication rate, incubation periods) peak at intermediate temperatures and decline at thermal extremes (30, 58). The largely positive temperature–risk relationships observed in our synthesis, especially across vectored diseases (Fig. 4), could either reflect studies conducted below these thermal optima or be methodological artifacts of using linear models. Ignoring these biologically grounded nonlinear dynamics can overestimate risks in warm regions near thermal limits and underestimate them in cooler regions, and—where thresholds are already met—can make climate-sensitive systems appear unresponsive in observational analyses (30, 55–57). Future research should consistently incorporate nonlinear models informed by vector-pathogen thermal biology to better capture the complex climate–disease dynamics.

The methodological limitations extend beyond the lack of nonlinear models to encompass other complex relationships in climate–disease dynamics. Most studies did not investigate seasonal dependencies (only 5.7% used seasonal autoregressive models that can capture periodic patterns), and while methods such as DLNMs and GAMs directly address nonlinear and lagged effects, they represent a small fraction of the dataset (5.2% and 4.8% of source studies, respectively). Although over half of the source studies used generalized linear or mixed-effects models (GL(M)Ms) theoretically capable of capturing complex interactions, we saw that 80% of these employed either standard GLMs or linear models primarily testing main effects alone. More sophisticated approaches, such as mixed-effects models (9.4%), GEEs (2.3%), or Bayesian frameworks (2.3%), which could better accommodate hierarchical structures or complex dependencies, were rarely utilized. These complex ecological interactions, where precipitation might amplify temperature effects, for example, or seasonal timing might determine the magnitude of climate impacts on disease risk, remain largely unexplored. This analytical simplification likely contributes to the heterogeneity in observed effect sizes and may mask essential mechanisms through which climate influences disease dynamics.

It is also important to consider that most of the studies in our synthesis were conducted over relatively short time periods, with half of the study periods of 11 y or shorter, and 75% of studies lasting 15 y or less. Such timeframes predominantly capture yearly and medium-term variability, detecting transient responses to weather anomalies, rather than the prolonged, cumulative impacts of sustained climate change. While extrapolating these short- to medium-term climate-sensitivity trends into the future can help identify a range of possible disease risk scenarios, it may fail to account for the more complex ecological reorganization under persistent climate change. Over longer periods, sustained changes in rainfall and temperature can trigger shifts in community composition, for example, facilitating the establishment of predator populations that might ultimately regulate rodent densities and, consequently, disease transmission (3). Extended datasets spanning several decades also show that responses to precipitation can be significantly lagged, influencing host community composition in a delayed yet profound manner as species dominance shifts over time (81). Taken together, this suggests that to anticipate better how zoonotic risks may evolve under sustained climate change, future studies should move beyond short-term associations and examine the cascading ecological mechanisms, such as trophic interactions, delayed density dependence, and regime shifts that mediate climate–disease relationships over decadal scales.

### Future Outlook.

Overall, our study highlights substantial heterogeneity in climate sensitivity across zoonotic diseases, as evident in the wide variation in effect sizes even within specific pathogens (as illustrated in Fig. 4 *B* and *D*). This heterogeneity across both vectored and nonvectored diseases complicates our ability to generalize findings and draw consistent conclusions about the impacts of climatic factors on zoonotic disease risk. However, it also highlights that climate sensitivity is a widespread phenomenon affecting a broad spectrum of zoonotic diseases, not just those transmitted by vectors. It remains unclear whether this variation represents true differences in disease responses to climate across different contexts or is an artifact of methodological inconsistencies and geographic sampling biases. This ambiguity highlights a critical gap in our understanding.

Given these challenges and considering that many climate-sensitive diseases are in areas consistently projected to experience significant temperature increases—and in some cases, precipitation—there is a pressing need for more scoping and standardized research approaches. Adopting consistent methodologies that account for nonlinear relationships and control for common epidemiological biases will be essential. Such approaches would enable us to distinguish genuine ecological patterns from statistical noise, improving our ability to predict the mechanisms, locations, and magnitudes of climate change impacts on zoonotic diseases. Enhancing the rigor and transparency of future studies is crucial for informing effective public health interventions and policy decisions in the face of a changing climate.

Looking ahead, real progress will come from integrative, transdisciplinary efforts that fuse fine-scale field ecology with predictive analytics and globally harmonized surveillance. For example, high-resolution remote sensing, which provides data on land use, hydrology, and vegetation change, could be coupled with newly developed portable genomic sequencing methods and analyzed using causal-inference frameworks to begin distinguishing correlation from mechanism. Embedding these tools in a network of long-term “One Health” observatories, distributed across contrasting biomes and socioeconomic contexts, would provide the continuous, standardized data streams that have underpinned significant advances in global fields like climatology. Such a platform would not only enable the early detection of climate-driven shifts in reservoir hosts or vectors but would also support probabilistic outbreak forecasting with explicit lead times applicable to public health services. Given the accelerating pace of warming, land conversion, and wildlife range shifts, building this infrastructure is not simply advisable—it is essential if we are to anticipate, rather than merely react to, the next wave of zoonotic threats.

## Materials and Methods

### Scoping Review of Literature.

We conducted a scoping literature search to identify peer-reviewed quantitative studies addressing the effect of climatic factors (i.e., temperature, precipitation, and humidity) on components contributing to overall zoonotic burden. The databases searched included PubMed (to capture healthcare-related studies narrowly) and Google Scholar (to ensure wide coverage of disciplines and journals), and the search was conducted primarily between November 15th and 22nd, 2022, with a supplementary search between August 11th and September 2nd, 2025. To ensure thoroughness, a list of 53 generic zoonotic disease names and their synonyms was compiled (*SI Appendix*, Table S2). Terms describing zoonotic burden/risk were chosen to encompass all potential stages where disease risk could be influenced (i.e., hazard, exposure, and vulnerability). Searches were structured by pairing a specific disease (e.g., Brucellosis) with “Climat*” as subject headings. “Climat* incorporated alternative keywords such as “Climate change,” “Climatic change,” “Climate sensitive,” or “Climate conditions.” To refine searches, Boolean operators were used to apply additional parameters (abundance/incidence/seroprevalence/cases) to search queries. Detailed search structures and example strings of search terms are provided in the *SI Appendix*, Table S1.

### Inclusion Criteria and Screening.

The inclusion criteria for this study were quantitative field studies directly evaluating correlations between climatic factors and metrics of zoonotic risk. The climatic parameters were defined as temperature, precipitation, and humidity. Eligible studies were identified through a three-step screening process: First, titles were reviewed, followed by abstracts, and then full texts (*SI Appendix*, Fig. S1). For each search query, the first 50 returned titles (ranked by the default “relevance” sorting algorithm in both Google Scholar and PubMed) were reviewed against the inclusion criteria. As eight searches were performed for each disease (see log of literature search in Dataset S1), reviewing the top 50 titles per search gathered an exhaustive list of titles while reducing literature screening time. Following the removal of duplicate entries, abstracts were further reviewed for relevance to the research question. Finally, a full-text review of relevant articles was completed to extract data on climatic factors and zoonotic burden/risk.

### Data Extraction and Effect Size Calculations.

Statistical values, associated sample sizes, and, when available, dispersion values (e.g., SE, SD, 95% CI) were extracted from compiled articles. We also extracted geographical data, including continent, country, locality, longitude, and latitude, of the study sites. Data presented in text or tables were directly extracted, and data from figures were digitized using WebPlotDigitizer (82). Data quality was assured by confirming interpretations (i.e., increases or decreases in zoonotic risk) with each study’s own assessment of described trends.

For this investigation, we defined a standardized measure of effect size using Hedges’ g (83). Observations were converted from presented statistics (i.e., odds ratio, relative risk, regression coefficient, correlation coefficient, t-, z-, f-, χ^2^ statistics) to Hedges’ g using standard conversion equations within the {esc} R package (84). The direction of the Hedges g value (i.e., a positive or negative value) determined whether a value represented an increase or decrease in zoonotic risk. To validate the robustness of conversion techniques, unit testing was performed using {tinytest} (85) and {testthat} packages (86) in R. Additionally, linear regressions were performed using the *lm* function from the {stats} package to measure the strength of correlations between obtained effect sizes and original statistical values (*SI Appendix*, Fig. S5).

Each study was categorized by the disease studied (e.g. Hantaviral diseases, Echinococcoses, Arenaviral diseases), risk/hazard metric (e.g. host disease incidence, host infection, host abundance), principal reservoir of the disease (e.g. rodents, mammals, livestock), pathogen type causing the disease (Virus, Bacteria, Parasite), type of the relationship between climate and disease risk (linear, nonlinear) and modeling method used (e.g. GL(M)M, Correlation, (S)ARIMA) (Fig. 2 and *SI Appendix*, Table S1). Additionally, diseases were categorized based on their mode of transmission into two groups: vectored and nonvectored diseases.

### Statistical Evaluation of Reported Direction Statements.

We used Pearson’s chi-squared test for count Data (from the {stats} R package) to investigate whether the proportions of reported increases and decreases in disease risk were significantly different. We performed bootstrap resampling with 1,000 iterations, sampling 80% of the data with replacement in each iteration. The 80% resampling was chosen to balance between reducing variance and maintaining sufficient sample size for reliable estimation. Specifically, we performed chi-squared tests on each bootstrap sample for the entire dataset and for the three climate subsets: temperature, precipitation, and humidity.

To ensure robustness, we repeated the procedure by sequentially removing records from one major group at a time (e.g., separately removing records from each of the top three diseases) from the analysis, to identify which categories significantly affected the proportions of increases to decreases in disease risk. Due to multiple comparisons across subsets of our data (e.g., transmission types, reservoirs, and pathogen types), we applied the Benjamini–Hochberg procedure to correct for multiple testing and control the false discovery rate.

### Analysis of Standardized Effect Sizes.

We categorized effect sizes into three groups based on the value of Hedges’ g: positive effect (g > 0.2), no effect (−0.2 < g < 0.2), and negative effect (g < −0.2). These thresholds were chosen based on Cohen’s conventions for small effect sizes (87), where values of 0.2 represent a small effect. We interpret the no effect category as no detected effect within the study’s model and the sampled climate range. Additionally, we analyzed the association between the reported direction of the climate impacts on disease risk (increase/decrease/no change) and the effect size categories based on our Hedges’ g calculations (negative effect/no effect/positive effect). To robustly estimate the significance of this association, we performed a bootstrap analysis with 1,000 samples. Each bootstrap sample consisted of 80% of the original data, sampled with replacement. We applied Fisher’s exact test to each bootstrap sample to determine the *P*-value for the association between the two variables.

To assess whether climate effects on measures of disease risk are uniform across different subsets of the data (specific transmission types, reservoirs, pathogens, vectors, countries, and diseases), we compared the effect size distribution for each subset to the full effect size distribution for each climate factor (temperature, precipitation, and humidity). We performed a two-sample permutation-based Anderson–Darling test with 1,000 replicates to compare the subset effect size distribution with the full distribution, testing whether they are likely to be from the same distribution. The Anderson–Darling test was chosen because it is a powerful nonparametric test that is sensitive to differences in both the location and shape of distributions, particularly in the tails. As one distribution is a subset of the other, we also performed the test by removing the subset from the full distribution.

We also compared the effect size distributions for vectored and nonvectored diseases using the Kolmogorov–Smirnov test to investigate whether the climate factors have the same effect on these groups. Unlike the two-sided Anderson–Darling test, the Kolmogorov–Smirnov statistic can be applied in a one-sided form, enabling us to test our directional hypothesis that effect sizes for vector-borne diseases would be larger (i.e., more positive) than those for nonvectored diseases. Given multiple comparisons among subsets (e.g., transmission types, reservoir hosts, vector types), we applied the Benjamini–Hochberg correction to *P*-values obtained from Anderson–Darling tests to limit the false discovery rate.

### Analysis of Projected Climatology Data across Study Sites.

To assess the co-occurrence of climate-sensitive diseases with areas expected to experience significant shifts in climatic conditions with future climate change, we extracted modeled climate shifts for study locations that reported positive (Hedges’ g > 0.2) or negative (Hedges’ g < −0.2) climate sensitivity effects.

To determine expected climate shifts, we used bioclimatic data from the CHELSA V2.1 CMIP6 dataset (88, 89), focusing on two climate variables: BIO1 (mean annual air temperature) and BIO12 (annual precipitation amount). We selected BIO1 and BIO12 as single, interpretable proxies because most extracted study metrics use mean/totals (temperature: 82% mean/median; precipitation: 62% totals + 17% mean/median), and at our study coordinates BIO1 and BIO12 were strongly aligned with the corresponding intra-annual mean/total layers (e.g., BIO1 with BIO11 r = 0.93, BIO10 r = 0.80; BIO12 with BIO16 r = 0.96, BIO13 r = 0.94; GFDL-ESM4, SSP3-7.0, 2041 to 2070), whereas seasonality/range layers showed weak or negative correlations and were rarely the reported study metric.

The BIO1 and BIO12 variables were extracted for both the baseline period (1981 to 2010) and a future time window (2041 to 2070) under three Shared Socioeconomic Pathways (SSP1-RCP2.6, SSP3-RCP7.0, SSP5-RCP8.5). We selected five GCMs (Global Climate Models) (GFDL-ESM4, IPSL-CM6A-LR, MPI-ESM1-2-HR, MRI-ESM2-0, UKESM1-0-LL) to capture variability across model predictions and to ensure a robust comparison across different climate scenarios.

For each spatial point corresponding to a study location, we extracted baseline and future BIO1 and BIO12 values from the climate rasters. This allowed us to compute the expected change in temperature (°C) and precipitation (mm) between the baseline and future projections. The differences were calculated for each combination of SSP and GCM, yielding 15 projected differences for each site to account for the variability and uncertainty inherent in climate projections and emission scenarios.

To capture the magnitude of climate shifts at each location, we categorized the temperature and precipitation differences based on a set of three temperature and three precipitation thresholds. For temperature, at each spatial point, we identified the proportion of SSP × GCM combinations predicting increases greater than or less than 1 °C, 1.5 °C, and 2 °C. Similarly, for precipitation, we calculated the proportion of SSP × GCM combinations predicting increases or decreases beyond ±25 mm, ±50 mm, and ±100 mm, and the proportions of combinations falling in the intermediate range (e.g., proportion of predictions > +25 mm, between –25 mm to +25 mm, and < –25 mm). Then, for each of the three temperature and precipitation thresholds, we summarized the modal categories of climate change for each location across the 15 SSP × GCM combinations, as there was generally strong agreement about the direction and magnitude of expected shifts.

Transforming continuous climate data into a set of discrete categories of temperature and precipitation changes enabled us to assess whether certain locations with positive (Hedges’ g > 0.2) or negative (Hedges’ g < −0.2) climate sensitivity effects are associated with a specific magnitude or direction of climate change. We constructed six contingency tables (for three temperature and three precipitation thresholds, see Fig. 5) and compared the association between disease climate sensitivity effects (positive/negative Hedges’ g) and the magnitude of predicted climate changes. Depending on the structure of the contingency table, either Pearson’s chi-squared test for count data (if all cells in the contingency table had five or more counts) or Fisher’s exact test for Count Data was applied to evaluate the association between these sensitivity categories and climate change magnitudes. We performed the tests on 1,000 bootstrap replicates for each contingency table and calculated the mean *P*-values, test statistics, 95% CI, and the percentage of iterations with a significant (*P* < 0.05) test result.

### Publication Bias Assessment.

To assess publication bias, we investigated the correlation between the OpenAlex (90) 2-y mean citedness index of journals included in our study and their respective Hedges’ g and *P*-values. We selected Pearson’s product-moment correlation test (from the {stats} package) to evaluate the correlations. Additionally, we applied the Interquartile Range (IQR) method to detect the outliers within the Hedges’ g and *P*-value data. Outliers were defined as values that fell more than 1.5 times the IQR below the first quartile or above the third quartile. The identified outliers included 122 of Hedges’ g values and 22 of *P*-values. To examine whether these outliers influenced the direction or significance of the correlations, we performed Pearson’s product-moment correlation tests twice: before and after removing the outliers (*SI Appendix*, Fig. S2).

Furthermore, we prepared funnel plots visualizing the distribution of effect sizes against their SE, where such calculations were possible. The funnel plots were created for the overall dataset and for the three climate factors (temperature, precipitation, and humidity). To each plot, we applied Egger’s regression test from the {metafor} package (91) to check for significant asymmetry, indicating potential publication bias. Egger’s test assesses whether there is a linear relationship between the effect sizes and their SE; a significant result suggests asymmetry in the funnel plot, which may be due to publication bias. To further investigate potential “p-hacking,” we visually inspected the distribution of *P*-values reported in the studies, focusing on the values surrounding *P* = 0.05.

## Supplementary Material

Appendix 01 (PDF)

Dataset S01 (XLSX)

## Data Availability

All parts of the analysis were conducted using R version 4.3.1. The full dataset and R code used to perform the analyses and create the figures have been deposited to Zenodo (10.5281/zenodo.15206104) (92) and GitHub (https://github.com/BioDivHealth/climate_meta).
