# Supplementary material for: Climate sensitivity is widely but unevenly spread across zoonotic diseases
Source: Proc Natl Acad Sci U S A. 2025 Dec 9;122(50):e2422851122. doi: 10.1073/pnas.2422851122 (PMC12718308; doi:10.1073/pnas.2422851122)
Supplement: Supplementary file 1 — Appendix 01 (PDF) [file pnas.2422851122.sapp.pdf]

PNAS

Supporting Information for

Climate sensitivity is widely but unevenly spread across zoonotic diseases

Artur Trebski[1‡], Lewis Gourlay[2‡], Rory Gibb[2], Natalie Imirzian[1], David W. Redding*[1,2]

[1] Natural History Museum, Cromwell Road, London, SW7 5BD, United Kingdom.

[2] Centre for Biodiversity and Environment Research, Department of Genetics, Evolution and Environment, University College London, Gower Street, London, WC1E 6BT, United Kingdom.

[‡] These authors contributed equally

* Corresponding author: David W. Redding

Email: david.redding@nhm.ac.uk

This PDF file includes:

Figures S1 to S5

Tables S1 to S9

Legends for Dataset S1

Extended Citations

Other supporting materials for this manuscript include the following:

Dataset S1

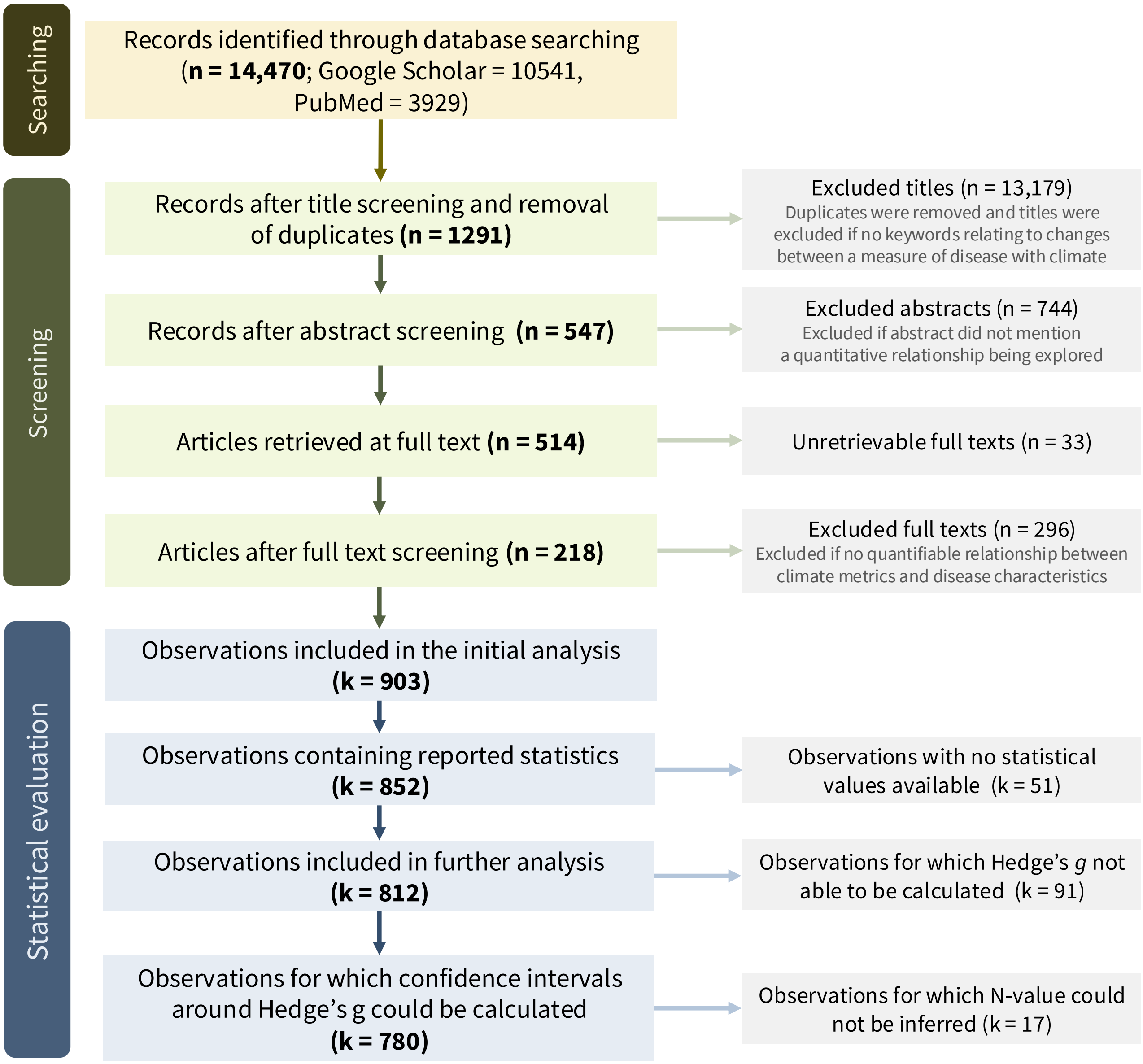

Fig. S1. Flow chart visualising the process of literature search, literature screening and statistical evaluation of extracted data. ***n*** values correspond to the number of scientific articles and ***k*** values correspond to the number of observations extracted.

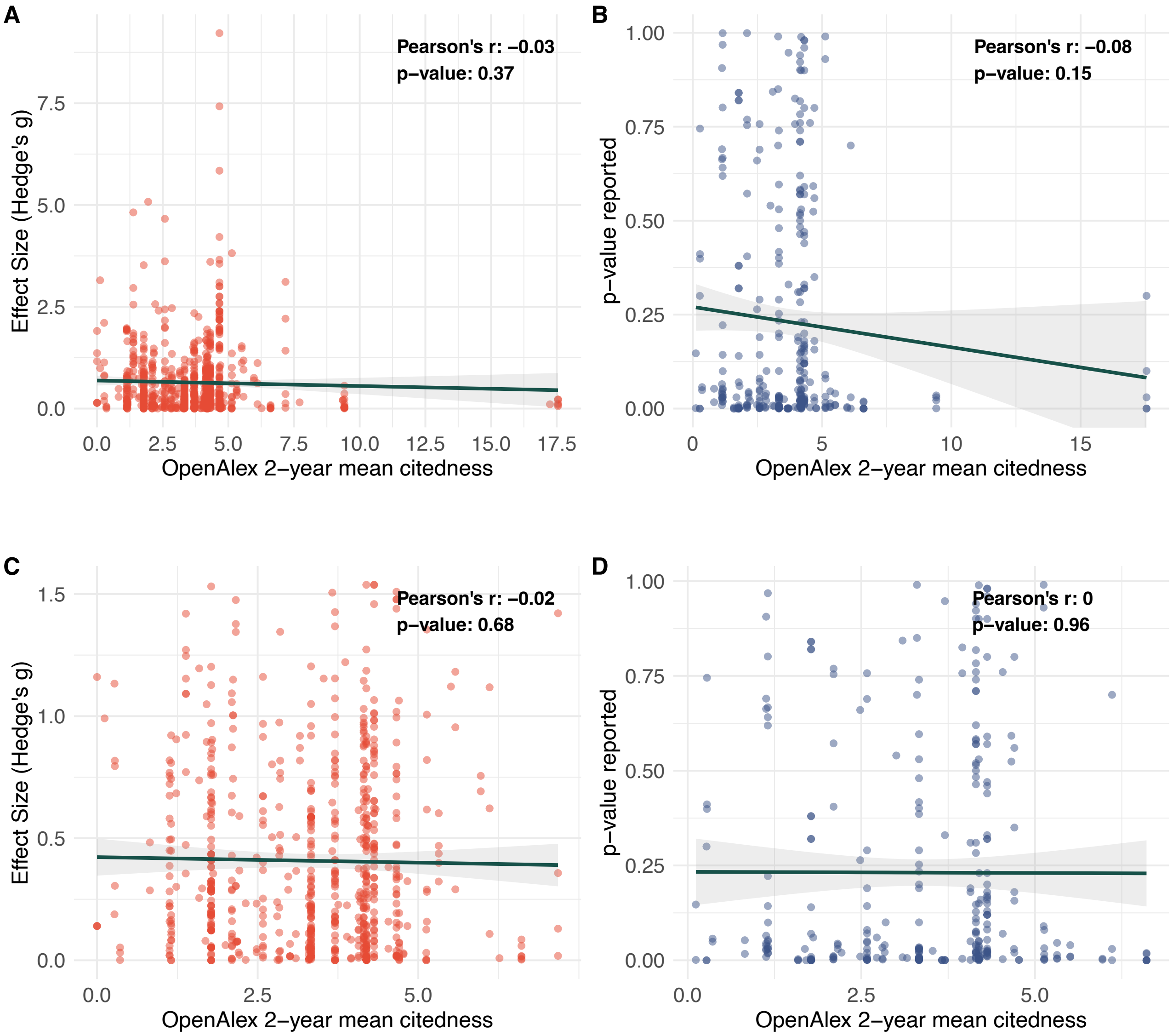

Fig. S2. Hedges' g effect sizes (A,C) and reported p-values (B,D) plotted against OpenAlex 2-year mean citedness of journals in which corresponding studies were published. Results of Pearson's product-moment correlation test (correlation coefficient and p-value) are annotated in the upper right corner of each subplot. Subplots A and B are based on full dataset and subplots C and D were made following removal of outliers among Hedges' g and p-value data using IQR method.

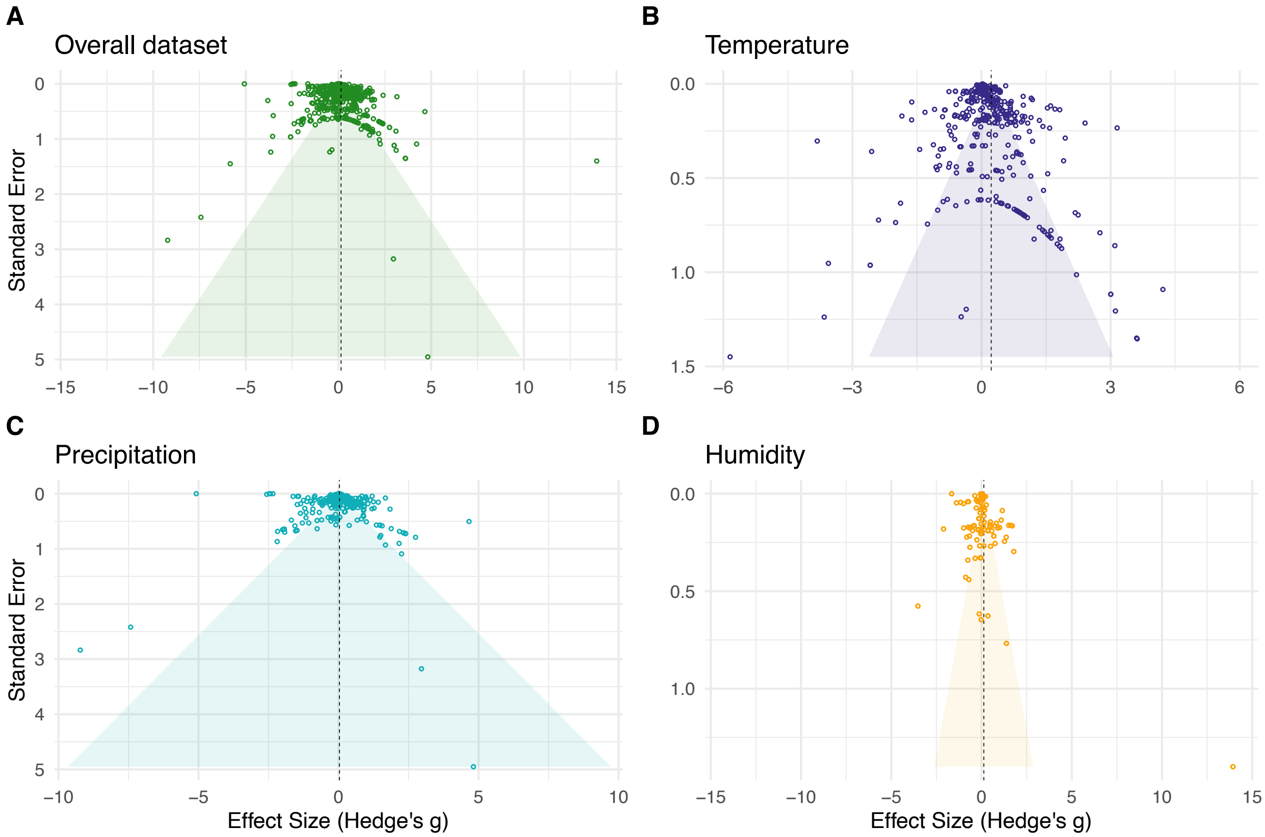

Fig. S3. Funnel plots of standard errors against corresponding Hedges' g values. Plot A made for the overall dataset, plots B, C and D made for Temperature, Precipitation and Humidity data respectively. Vertical dotted line represents the mean effect size within each plot. Shaded area represents 95% confidence region constructed for each plot.

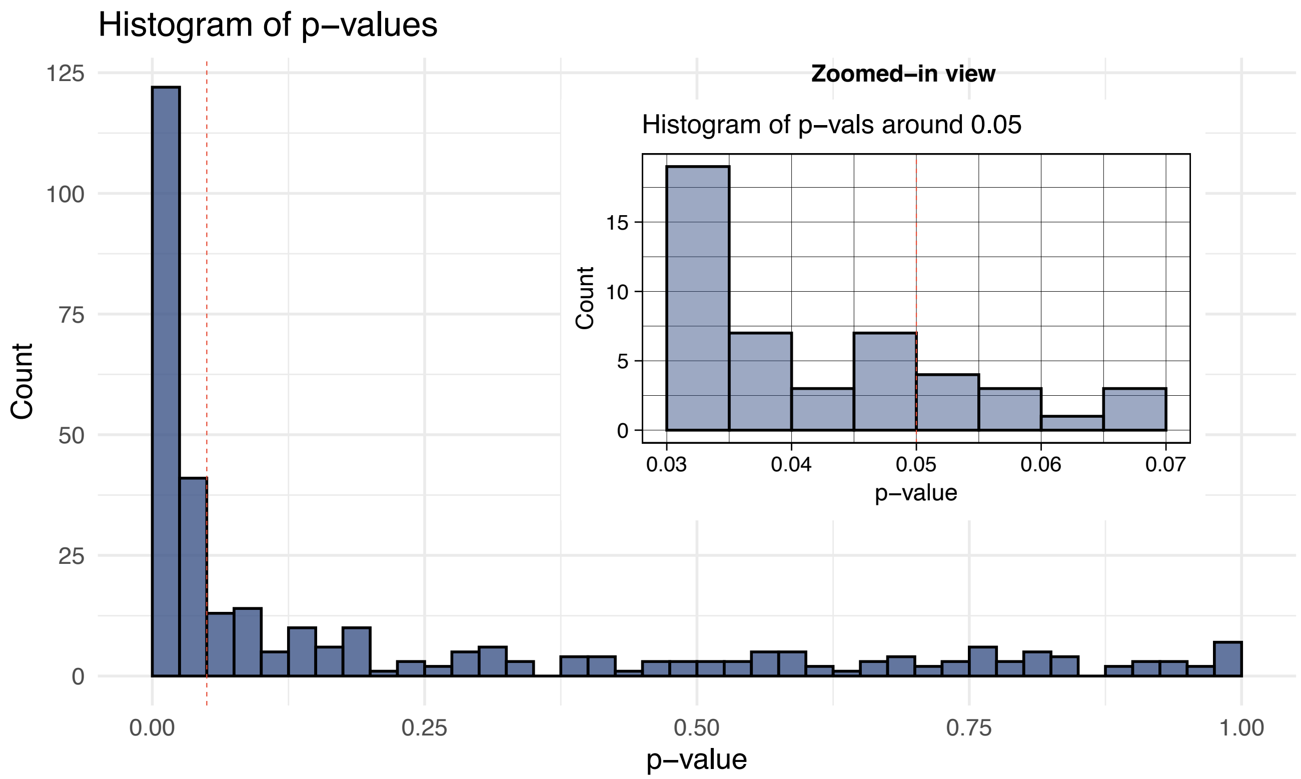

Fig. S4. Distribution of p-values in study sample. The main plot shows the overall distribution of p-values from all studies for which numeric p-values were provided. X-axis represents the p-values, and Y-axis represents the count of p-values falling within a specific bin. The inset plot shows the distribution of p-values where $0.03 < p < 0.07$. Red vertical lines signify p-value = 0.05.

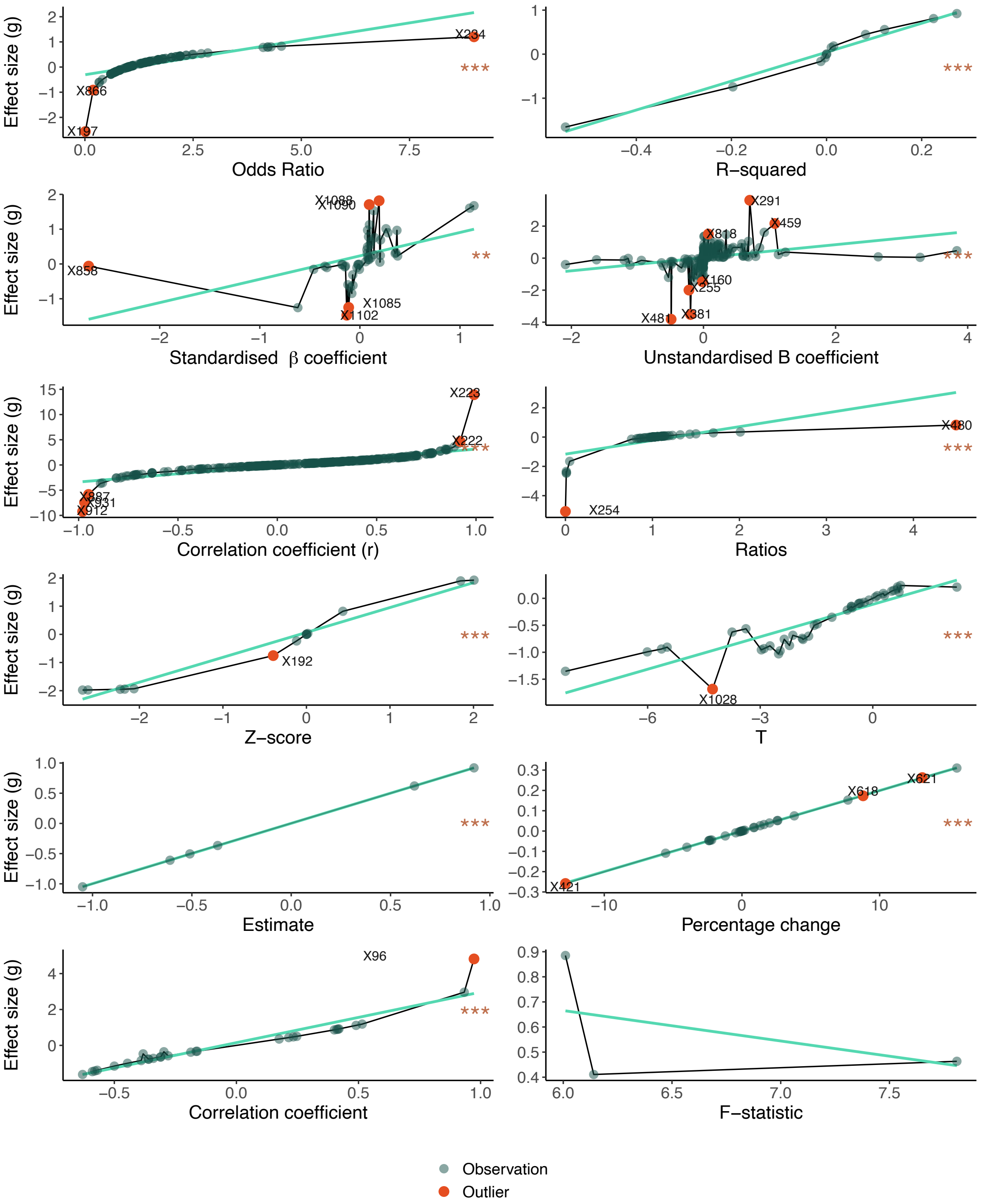

Fig. S5. Relationships between extracted statistical measures and corresponding Hedges' g values. The plots show the relationship between different statistical measures (e.g., Odds Ratio, Correlation Coefficient, F-statistic) sourced from studies and the calculated effect sizes (Hedges' g). Dark green points indicate individual observations and red points highlight outliers. Outliers were determined based on residuals from a linear regression model, where points with residuals greater than two standard deviations from the mean were marked as outliers. The light green line represents the linear fit for each measure.

Table S1. Table of the inclusion criteria, definitions and search structures used to identify relevant articles during systematic review. “DISEASE NAME” refers to the specific disease targeted by each search.

Criteria	Details	
**Databases used**	Google Scholar, PubMed	
**Inclusion criteria**	Studies highlighting an explicit relationship between zoonotic risk and climatic factors at an implicit place/region.	
**Climate change definition**	Mentions climate or meteorological variables either as an umbrella or specific term (e.g., temperature or spring temperature). Change defined as any alteration (either observed or projected) to climatic parameters.	
**Zoonotic risk definition**	Any contributory component of zoonotic (i.e., explicitly animal-borne) burden or risk. Primarily reservoir/pathogen abundance, seroprevalence, number of cases or incidence rates	
**Dates of literature search**	15th November – 22nd November 2022, with supplementary search conducted 11th August – 2nd September 2025	
**Languages**	English	
**Search structure**	**Search subject headings**	**Coupled terms**
**S1**	("*DISEASE NAME*" AND "Climat*")	AND "Abundance"
**S2**	("*DISEASE NAME*" AND "Climat*")	AND “Seroprevalence*"
**S3**	("*DISEASE NAME*" AND "Climat*")	AND "Cases" OR "Incidence"
**S4**	("*DISEASE NAME*" AND "Climat*")	AND "Abundance" OR "Seroprevalence"<br>OR "Cases" OR "Incidence"
**Example string of search terms**	((Japanese encephalitis) AND (Climat*))<br>AND ((Cases) OR (Incidence) OR (Abundance) OR (Seroprevalence))	

Table S2. Names of zoonotic diseases used in literature search. Each name replaced the "DISEASE NAME" field in the string of search terms presented in Table S1. In some cases, additional synonyms of the disease names were used, these can be found in Supplementary File 1, along with complete search structures.

Pathogen	Transmission	Disease	Studies	Metrics
Bacteria	Non-vectored	Anthrax	3	5
		Brucellosis	17	66
		Campylobacteriosis	1	1
		Canine Leptospirosis	1	2
		Leptospirosis	23	59
		Q-fever	3	11
		Salmonellosis	1	1
	Vectored	Anaplasmosis	1	1
		Bartonellosis	1	1
		Borreliosis	1	3
		Lyme disease	1	1
		Mediterranean spotted fever	1	1
		Murine typhus	2	4
		Plague	8	13
		Rickettsial disease	2	6
		Rocky Mountain Spotted Fever	1	4
		Scrub typhus	12	59
		Typhus group rickettsiosis	1	2
Parasite	Non-vectored	Alveolar echinococcosis	6	13
		Cryptosporidiosis	4	8
		Cystic echinococcosis	4	9
		Giardiasis	1	2
		Human echinococcosis	1	2
		Talaromycosis	1	2
		Toxoplasmosis	1	2
	Vectored	Theileriosis	1	1
Virus	Non-vectored	Andes virus	2	4
		Anjozorobe virus	1	3
		Avian Influenza	2	11
		Dobrava-Belgrade virus	1	2
		Ebola	1	2
		Haemorrhagic fever with renal syndrome	28	90
		Hantaan virus	1	2
		Hantavirus pulmonary syndrome	4	13
		Hendra virus	3	6
		Junin virus	1	2
		Lassa fever	2	6
		MERS-CoV	1	2
		Monkeypox	1	1
		Nephropathia epidemica	3	11
		Nipah virus	3	6
		Puumala virus	5	14
		Rabies	5	11
		Seoul hantavirus	1	3
		Sin Nombre Virus	2	2
	Vectored	Crimean congo haemorrhagic fever	4	20
		Japanese encephalitis	17	53
		Rift valley fever	4	14
		Yellow Fever	4	18
		Tick-borne encephalitis	8	17
		West Nile Virus	29	261

Table S3. Results of Pearson's Chi-squared Test for Count Data conducted on the data describing reported direction (increase vs decrease) of the impacts of all climate effects on disease risk for the overall dataset. The chi-squared goodness-of-fit tests assessed whether the observed frequencies of reported increases and decreases in disease risk significantly differed from the expected frequencies under the null hypothesis (i.e., no difference in the likelihood of increases and decreases). First row shows results of chi-square tests using the full dataset. Subsequent rows report the results of tests in which major diseases, countries, reservoirs, pathogen types, statistical methods and transmission types were removed from the analyses one-at-a-time. P-values are provided both as raw values and adjusted using Benjamini–Hochberg correction. Significant results suggest an uneven proportion of increases to decreases of disease risk reported. Chi-square tests were run on 1000 bootstraps of 80% of the data. * = p < 0.05; ** = p < 0.01.

	Group removed	Chi-square statistic	Lower CI	Upper CI	P-value (raw)	P-value (adj.)	
**No groups dropped**	-	32.94	32.24	33.65	0.000	0.000	***
**Disease removed**	Hantaviral diseases	27.08	26.47	27.68	0.000	0.000	***
	Arboviral diseases	11.59	11.20	11.98	0.018	0.021	*
	Leptospirosis	33.59	32.91	34.28	0.000	0.000	***
**Country removed**	China	26.02	25.43	26.62	0.000	0.000	***
	Iran	31.86	31.19	32.53	0.000	0.000	***
	USA	39.66	38.94	40.37	0.000	0.000	**
**Principle reservoir removed**	Rodents	27.97	27.36	28.58	0.000	0.000	***
	Mammals (multispecies)	21.07	20.53	21.61	0.001	0.002	**
	Birds	9.78	9.41	10.15	0.031	0.035	*
**Pathogen type removed**	Virus	8.75	8.39	9.10	0.045	0.047	*
	Bacteria	29.07	28.41	29.73	0.000	0.000	***
	Parasite	29.85	29.22	30.47	0.000	0.000	***
**Statistical method removed**	Spearman rank correlation	19.13	18.60	19.66	0.003	0.004	**
	Pearson correlation	28.01	27.40	28.63	0.000	0.000	***
	Negative binomial regression	30.19	29.57	30.81	0.000	0.000	***
**Transmission type removed**	Non vectored	39.46	38.73	40.18	0.000	0.000	***
	Vectored	4.14	3.91	4.37	0.185	0.185	>0.05

Table S4. Results of Permutation-based Anderson-Darling test of homogeneity evaluating whether the distribution of effect sizes within specific groups (e.g., transmission types, principal reservoirs, pathogen types, or vector types) significantly differs from the overall distribution of effect sizes for <u>temperature</u>. The table compares the effect size distributions within a given group to the full temperature dataset and to the full temperature dataset with that specific group excluded. p-values and AD statistics are provided for both comparisons, with asterisks indicating significance levels (* = p < 0.05; ** = p < 0.01). P-values are provided both as raw values and adjusted using Benjamini–Hochberg correction. Sample sizes of specific groups are provided in columns after the name of the analysed group, with the first number representing the number of effect sizes and second representing the number of studies from which these effects were sourced.

Subsets of the dataset analysed				Group vs. full dataset			Group vs. full dataset excluding the group		
**Category**	**Group analysed**	**Nº effects**	**Nº studies**	**p-value (raw)**	**p-value (adj.)**	**AD stat.**	**p-value (raw)**	**p-value (adj.)**	**AD stat.**
**Transmission type**	*Vectored*	226	67	0.039	0.082	2.850	0.000	0.000 ***	11.122
	Non-vectored	154	95	0.006	0.016	4.705	0.000	0.000 ***	11.122
**Principal reservoir**	*Rodents*	93	64	0.005	0.016	4.183	0.002	0.005 **	6.809
	Mammals (multispecies)	83	40	0.936	0.936	0.298	0.748	0.748	0.500
	Livestock	37	21	0.044	0.084	2.589	0.020	0.032 *	3.190
	Birds	160	35	0.003	0.014	5.320	0.000	0.000 ***	12.768
**Pathogen**	*Virus*	264	102	0.797	0.841	0.463	0.050	0.068	2.554
	Bacteria	99	51	0.362	0.465	1.002	0.154	0.183	1.686
**Vector**	*Mosquito*	164	37	0.001	0.010	5.369	0.014	0.027 *	3.522
	Tick	36	15	0.367	0.465	0.992	0.006	0.013 *	3.959
**Country**	*China*	56	40	0.780	0.841	0.466	0.664	0.701	0.591
	Iran	22	12	0.059	0.102	2.397	0.031	0.045 *	2.710
	USA	75	11	0.078	0.118	2.250	0.019	0.032 *	3.173
	Northern Arctic	33	1	0.005	0.016	4.854	0.000	0.000 ***	6.501
	Russia	39	4	0	0.000	12.332	0.000	0.000 ***	15.671
**Disease**	*HFRS*	25	21	0.612	0.727	0.632	0.587	0.656	0.698
	Brucellosis	24	13	0.081	0.118	2.177	0.058	0.073	2.490
	West Nile Virus	137	19	0.007	0.017	5.178	0.000	0.000 ***	10.869
	Leptospirosis	27	17	0.002	0.013	5.189	0.000	0.000 ***	5.901

Table S5. Results of the Exact two-sample Kolmogorov-Smirnov Tests comparing the distribution of Effect Sizes (Hedges' g) between Vectored and Non-vectored diseases for different environmental conditions. The table included the test statistics and corresponding p-values for differences in effect size distributions between vectored and non-vectored transmission types under three environmental conditions: Temperature, Precipitation, and Humidity. A significant p-value suggests a difference between the distributions of the two groups. The test statistic represents the maximum difference between the empirical cumulative distribution functions (ECDFs) of the two groups.

Variable	Transmission type	Nº effects	Mean Hedges' g	SD Hedges' g	p-value	D^+ Test statistic
**Temperature**	Vectored	226	0.36	0.88	0.000 **	0.28
	Non-vectored	154	0.01	1.13		
**Precipitation**	Vectored	155	0.00	1.08	0.377	0.08
	Non-vectored	147	0.02	1.34		
**Humidity**	Vectored	56	0.05	0.70	0.501	0.10
	Non-vectored	55	0.20	2.07		

Table S6. Results of Permutation-based Anderson-Darling test of homogeneity evaluating whether the distribution of effect sizes within specific groups (e.g., transmission types, principal reservoirs, pathogen types, or vector types) significantly differs from the overall distribution of effect sizes for humidity. The table compares the effect size distributions within a given group to the full humidity dataset and to the full humidity dataset with that specific group excluded. P-values and AD statistics are provided for both comparisons, with asterisks indicating significance levels (* = p < 0.05; ** = p < 0.01). P-values are provided both as raw values and adjusted using Benjamini–Hochberg correction. Sample sizes of specific groups are provided in columns after the name of the analysed group, with the first number representing the number of effect sizes and second representing the number of studies from which these effects were sourced.

Subsets of the dataset analysed				Group vs. full dataset			Group vs. full dataset excluding the group		
**Category**	**Group analysed**	**Nº effects**	**Nº studies**	**p-value (raw)**	**p-value (adj.)**	**AD stat.**	**p-value (raw)**	**p-value (adj.)**	**AD stat.**
**Transmission type**	*Vectored*	56	26	1	1.000	0.136	0.846	0.853	0.413
	Non-vectored	55	41	0.998	1.000	0.141	0.853	0.853	0.413
**Principal reservoir**	*Rodents*	31	26	0.482	0.964	0.770	0.218	0.430	1.306
	Mammals (multispecies)	28	15	0.084	0.470	2.156	0.015	0.053	3.537
	Livestock	22	11	0.094	0.470	2.074	0.016	0.053	3.313
	Birds	27	12	0.454	0.964	0.831	0.236	0.430	1.301
**Pathogen**	*Virus*	60	38	0.885	1.000	0.385	0.258	0.430	1.196
	Bacteria	47	25	0.94	1.000	0.307	0.473	0.591	0.850
**Vector**	*Mosquito*	30	13	0.275	0.917	1.154	0.012	0.053	3.814
**Country**	*China*	34	27	0.653	1.000	0.605	0.337	0.481	1.049

Table S7. Results of Permutation-based Anderson-Darling test of homogeneity evaluating whether the distribution of effect sizes within specific groups (e.g., transmission types, principal reservoirs, pathogen types, or vector types) significantly differs from the overall distribution of effect sizes for <u>precipitation</u>. The table compares the effect size distributions within a given group to the full precipitation dataset and to the full dataset with that specific group excluded. p-values and AD statistics are provided for both comparisons, with asterisks indicating significance levels (* = p < 0.05; ** = p < 0.01). P-values are provided both as raw values and adjusted using Benjamini–Hochberg correction. Sample sizes of specific groups are provided in columns after the name of the analysed group, with the first number representing the number of effect sizes and second representing the number of studies from which these effects were sourced.

Subsets of the dataset analysed				Group vs. full dataset			Group vs. full dataset excluding the group		
**Category**	**Group analysed**	**Nº effects**	**Nº studies**	**p-value (raw)**	**p-value (adj.)**	**AD stat.**	**p-value (raw)**	**p-value (adj.)**	**AD stat.**
**Transmission type**	*Vectored*	155	55	0.641	0.808	0.605	0.101	0.165	1.776
	Non-vectored	147	90	0.665	0.808	0.581	0.120	0.180	1.776
**Principal reservoir**	*Rodents*	93	62	0.382	0.625	0.946	0.096	0.165	1.821
	Mammals (multispecies)	69	33	0.262	0.472	1.229	0.072	0.165	2.152
	Livestock	37	22	0.117	0.453	1.919	0.055	0.165	2.431
	Birds	99	27	0.157	0.453	1.644	0.012	0.054	3.038
**Pathogen**	*Virus*	183	82	0.802	0.849	0.419	0.150	0.193	1.593
	Bacteria	103	53	0.919	0.919	0.318	0.588	0.623	0.680
**Vector**	*Mosquito*	105	32	0.176	0.453	1.567	0.045	0.162	2.574
	Tick	25	8	0.249	0.472	1.183	0.079	0.165	2.075
**Country**	*China*	54	39	0.673	0.808	0.590	0.466	0.559	0.839
	USA	66	12	0.011	0.099	3.513	0.001	0.009 **	5.171
	Northern Arctic Region	33	1	0.001	0.018	6.953	0.000	0.000 ***	9.839
**Disease**	*HFRS*	24	21	0.595	0.808	0.665	0.519	0.584	0.746
	Brucellosis	21	12	0.202	0.455	1.399	0.150	0.193	1.629
	Leptospirosis	31	19	0.167	0.453	1.523	0.088	0.165	1.888
	Japanese encephalitis	22	14	0.77	0.849	0.477	0.689	0.689	0.554
	West Nile Virus	73	12	0.034	0.204	2.700	0.004	0.024 *	4.233

Table S8. Results of Pearson's Chi-squared Tests for Count Data and Fisher's Exact Tests for Count Data conducted on the data describing associations between climate sensitivity (Positive/Negative Hedges' g) and projected changes in temperature and precipitation across study sites. The tests were performed at three temperature thresholds (+1ºC, +1.5ºC, +2ºC) and three precipitation thresholds (±25 mm, ±50 mm, ±100 mm) using predictions from three SSP scenarios and five GCMs for the period 2041–2070 compared to baseline conditions (1981–2010). The p-values, 95% confidence intervals around p-values, and test statistics are reported. Tests were run on 1000 bootstrap samples and the percentage of tests with p-value under 0.05 is reported.

Variable	Threshold	Test	p-value	p-value 95% CI	Percentage of p-values < 0.05	Test statistic
**Temperature**	+1ºC	Chi-Square	0	-	100%	31.21
	+1.5ºC	Fisher's Exact	0.30	0.28 – 0.32	28%	–
	+2ºC	Chi-Square	0.56	0.54 – 0.58	5.3%	0.95
**Precipitation**	± 25mm	Fisher's Exact	0.13	0.12 – 0.14	52%	6.79
	± 50mm	Fisher's Exact	0.24	0.22 – 0.25	25.8%	–
	± 100mm	Fisher's Exact	0.19	0.18 – 0.21	39.8%	–

Table S9. Results of the Egger's regression tests on funnel plot asymmetry. This table presents the results of Egger's regression tests (***regtest {metafor}***) for funnel plot asymmetry, based on effect sizes and standard errors across the overall dataset and three climatic subsets: temperature, precipitation, and humidity. The z-value tests the null hypothesis of no funnel plot asymmetry, and the p-value indicates statistical significance. Significant p-values ($p < 0.05$) suggest publication bias.

Data	Intercept	Z-value	p-value
**Overall dataset**	-0.014	4.804	0.00
**Temperature**	0.082	3.078	0.002
**Precipitation**	-0.046	1.935	0.053
**Humidity**	-0.271	3.838	0.000

Dataset S1 (separate file).

Dataset S1 has been deposited in Zenodo (https://doi.org/10.5281/zenodo.15206104) as described in **Data, Materials, and Software Availability** section of the main manuscript.

`Dataset_S1.xlsx` (also available as separate CSV files) contains four sheets:

- `Metadata`: describes column names of the main dataset
- `Dataset`: main dataset containing data extracted from source studies and standardized effect sizes
- `Studies`: List of source studies with their titles, author lists, citations, URLs and DOIs.
- `Literature_Search_Log`: detailed log of literature search

Extended Citations. Below is the list of citations associated with 218 source studies included in this scoping review.

1. B. I. Kim, *et al.*, A Conceptual Model for the Impact of Climate Change on Fox Rabies in Alaska, 1980–2010. *Zoonoses and Public Health* **61**, 72–80 (2014).

2. K. S. Kreppel, *et al.*, A Non-Stationary Relationship between Global Climate Phenomena and Human Plague Incidence in Madagascar. *PLoS Negl Trop Dis* **8**, e3155 (2014).

3. M. Dadar, Y. Shahali, Y. Fakhri, A primary investigation of the relation between the incidence of brucellosis and climatic factors in Iran. *Microbial Pathogenesis* **139**, 103858 (2020).

4. G. Marini, *et al.*, A quantitative comparison of West Nile virus incidence from 2013 to 2018 in Emilia-Romagna, Italy. *PLOS Neglected Tropical Diseases* **14**, e0007953 (2020).

5. T. Roberts, *et al.*, A spatio-temporal analysis of scrub typhus and murine typhus in Laos; implications from changing landscapes and climate. *PLoS Negl Trop Dis* **15**, e0009685 (2021).

6. S. S. Han, S. Kim, Y. Choi, S. Kim, Y. S. Kim, Air pollution and hemorrhagic fever with renal syndrome in South Korea: an ecological correlation study. *BMC Public Health* **13**, 347 (2013).

7. Q. Li, *et al.*, Analysis of Incidence and Related Factors of Hemorrhagic Fever with Renal Syndrome in Hebei Province, China. *PLoS ONE* **9**, e101348 (2014).

8. R. Zhang, *et al.*, Analysis of the effect of meteorological factors on hemorrhagic fever with renal syndrome in Taizhou City, China, 2008–2020. *BMC Public Health* **22**, 1097 (2022).

9. H. Xiao, *et al.*, Animal Reservoir, Natural and Socioeconomic Variations and the Transmission of Hemorrhagic Fever with Renal Syndrome in Chenzhou, China, 2006–2010. *PLoS Negl Trop Dis* **8**, e2615 (2014).

10. H. E. Brown, *et al.*, Annual Seroprevalence of *Yersinia pestis* in Coyotes as Predictors of Interannual Variation in Reports of Human Plague Cases in Arizona, United States. *Vector-Borne and Zoonotic Diseases* **11**, 1439–1446 (2011).

11. S. E. Hutter, *et al.*, Assessing changing weather and the El Niño Southern Oscillation impacts on cattle rabies outbreaks and mortality in Costa Rica (1985–2016). *BMC Vet Res* **14**, 285 (2018).

12. G. Paternoster, *et al.*, Association between environmental and climatic risk factors and the spatial distribution of cystic and alveolar echinococcosis in Kyrgyzstan. *PLoS Negl Trop Dis* **15**, e0009498 (2021).

13. L.-Y. Huang, *et al.*, Association between Hemorrhagic Fever with Renal Syndrome Epidemic and Climate Factors in Heilongjiang Province, China. *The American Journal of Tropical Medicine and Hygiene* **89**, 1006–1012 (2013).

14. T. Tu, *et al.*, Association between meteorological factors and the prevalence dynamics of Japanese encephalitis. *PLoS ONE* **16**, e0247980 (2021).

15. J. Borah, P. Dutta, S. A. Khan, J. Mahanta, Association of Weather and Anthropogenic Factors for Transmission of Japanese Encephalitis in an Endemic Area of India. *EcoHealth* **10**, 129–136 (2013).

16. B. K. Chhetri, *et al.*, Associations between extreme precipitation and acute gastro-intestinal illness due to cryptosporidiosis and giardiasis in an urban Canadian drinking water system (1997–2009). *Journal of Water and Health* **15**, 898–907 (2017).

17. H. Xiao, *et al.*, Atmospheric Moisture Variability and Transmission of Hemorrhagic Fever with Renal Syndrome in Changsha City, Mainland China, 1991–2010. *PLoS Negl Trop Dis* **7**, e2260 (2013).

18. M. Ferenczi, *et al.*, Avian influenza infection dynamics under variable climatic conditions, viral prevalence is rainfall driven in waterfowl from temperate, south-east Australia. *Vet Res* **47**, 23 (2016).

19. J. L. Orrock, B. F. Allan, C. A. Drost, Biogeographic and Ecological Regulation of Disease: Prevalence of Sin Nombre Virus in Island Mice Is Related to Island Area, Precipitation, and Predator Richness. *The American Naturalist* **177**, 691–697 (2011).

20. H.-Y. Tian, *et al.*, Changes in Rodent Abundance and Weather Conditions Potentially Drive Hemorrhagic Fever with Renal Syndrome Outbreaks in Xi'an, China, 2005–2012. *PLoS Negl Trop Dis* **9**, e0003530 (2015).

21. J. Lourenço, R. N. Thompson, J. Thézé, U. Obolski, Characterising West Nile virus epidemiology in Israel using a transmission suitability index. *Eurosurveillance* **25** (2020).

22. M. Miterpáková, P. Dubinský, K. Reiterová, M. Stanko, CLIMATE AND ENVIRONMENTAL FACTORS INFLUENCING ECHINOCOCCUS MULTILOCULARIS OCCURRENCE IN THE SLOVAK REPUBLIC. *Ann Agric Environ Med.* **13**, 235–242 (2006).

23. A. Latinne, S. Morand, Climate Anomalies and Spillover of Bat-Borne Viral Diseases in the Asia–Pacific Region and the Arabian Peninsula. *Viruses* **14**, 1100 (2022).

24. S. Sipari, *et al.*, Climate change accelerates winter transmission of a zoonotic pathogen. *Ambio* **51**, 508–517 (2022).

25. A. Anyamba, *et al.*, Climate Conditions During a Rift Valley Fever Post-epizootic Period in Free State, South Africa, 2014–2019. *Front. Vet. Sci.* **8**, 730424 (2022).

26. W. S. D. Tennant, M. J. Tildesley, S. E. F. Spencer, M. J. Keeling, Climate drivers of plague epidemiology in British India, 1898–1949. *Proc. R. Soc. B.* **287**, 20200538 (2020).

27. F. Ding, *et al.*, Climate drives the spatiotemporal dynamics of scrub typhus in China. *Global Change Biology* **28**, 6618–6628 (2022).

28. A. Altamimi, A. E. Ahmed, Climate factors and incidence of Middle East respiratory syndrome coronavirus. *Journal of Infection and Public Health* **13**, 704–708 (2020).

29. Y. Gao, *et al.*, Climate factors driven typhus group rickettsiosis incidence dynamics in Xishuangbanna Dai autonomous prefecture of Yunnan province in China, 2005–2017. *Environ Health* **19**, 3 (2020).

30. R. J. Eisen, *et al.*, Climate Predictors of the Spatial Distribution of Human Plague Cases in the West Nile Region of Uganda. *The American Journal of Tropical Medicine and Hygiene* **86**, 514–523 (2012).

31. W.-Y. Zhang, *et al.*, Climate Variability and Hemorrhagic Fever with Renal Syndrome Transmission in Northeastern China. *Environ Health Perspect* **118**, 915–920 (2010).

32. P. Bi, S. Tong, K. Donald, K. A. Parton, J. Ni, Climate Variability and Transmission of Japanese Encephalitis in Eastern China. *Vector-Borne and Zoonotic Diseases* **3**, 111–115 (2003).

33. Y. Wei, *et al.*, Climate variability, animal reservoir and transmission of scrub typhus in Southern China. *PLoS Negl Trop Dis* **11**, e0005447 (2017).

34. P. W. Dhewantara, *et al.*, Climate variability, satellite-derived physical environmental data and human leptospirosis: A retrospective ecological study in China. *Environmental Research* **176**, 108523 (2019).

35. A. Lal, T. Ikeda, N. French, M. G. Baker, S. Hales, Climate Variability, Weather and Enteric Disease Incidence in New Zealand: Time Series Analysis. *PLoS ONE* **8**, e83484 (2013).

36. M. Lukan, E. Bullova, B. Petko, Climate Warming and Tick-borne Encephalitis, Slovakia. *Emerg. Infect. Dis.* **16**, 524–526 (2010).

37. L. T. Savage, R. M. Reich, L. M. Hartley, P. Stapp, M. F. Antolin, Climate, soils, and connectivity predict plague epizootics in black-tailed prairie dogs (*Cynomys ludovicianus*). *Ecological Applications* **21**, 2933–2943 (2011).

38. P. Pravin, K. Praveen, S. P. Parth, D. Pradeep, Climatic Determinants of Japanese Encephalitis in Bihar State of India: A Time-Series Poisson Regression Analysis. *JCD* **49**, 13–18 (2018).

39. E. S. Hatton, *et al.*, Climatic Predictors of the Intra- and Inter-Annual Distributions of Plague Cases in New Mexico Based on 29 Years of Animal-Based Surveillance Data. *The American Journal of Tropical Medicine and Hygiene* **82**, 95–102 (2010).

40. P. Bi, S. Tong, K. Donald, K. Parton, J. Ni, Climatic, reservoir and occupational variables and the transmission of haemorrhagic fever with renal syndrome in China. *International Journal of Epidemiology* **31**, 189–193 (2002).

41. D. J. Páez, *et al.*, Conditions affecting the timing and magnitude of Hendra virus shedding across pteropodid bat populations in Australia. *Epidemiol. Infect.* **145**, 3143–3153 (2017).

42. M. Daniel, B. Kříž, V. Danielová, J. Valter, I. Kott, Correlation between meteorological factors and tick-borne encephalitis incidence in the Czech Republic. *Parasitol Res* **103**, 97–107 (2008).

43. H. Ansari, *et al.*, Crimean-Congo hemorrhagic fever and its relationship with climate factors in southeast Iran: a 13-year experience. *J Infect Dev Ctries* **8**, 749–757 (2014).

44. A. Tran, *et al.*, Describing fine spatiotemporal dynamics of rat fleas in an insular ecosystem enlightens abiotic drivers of murine typhus incidence in humans. *PLoS Negl Trop Dis* **15**, e0009029 (2021).

45. F. Polop, M. C. Provensal, J. Priotto, A. Steinmann, J. J. Polop, Differential effects of climate, environment, and land use on two sympatric species of *Akodon*. *Studies on Neotropical Fauna and Environment* **47**, 147–156 (2012).

46. I. Fischer, *et al.*, Distribution of alveolar echinococcosis according to environmental and geographical factors in Germany, 1992-2018. *Acta Tropica* **212**, 105654 (2020).

47. P. Giraudoux, *et al.*, Drivers of Echinococcus multilocularis Transmission in China: Small Mammal Diversity, Landscape or Climate? *PLoS Negl Trop Dis* **7**, e2045 (2013).

48. R. Peng, *et al.*, Driving effect of multiplex factors on human brucellosis in high incidence region, implication for brucellosis based on one health concept. *One Health* **15**, 100449 (2022).

49. S. H. Paull, *et al.*, Drought and immunity determine the intensity of West Nile virus epidemics and climate change impacts. *Proc Biol Sci* **284** (2017).

50. G. Wang, R. B. Minnis, J. L. Belant, C. L. Wax, Dry weather induces outbreaks of human West Nile virus infections. *BMC Infect Dis* **10**, 38 (2010).

51. D. Gikungu, *et al.*, Dynamic risk model for Rift Valley fever outbreaks in Kenya based on climate and disease outbreak data. *Geospat Health* **11** (2016).

52. A. Simon, *et al.*, Ecology of Arctic rabies: 60 years of disease surveillance in the warming climate of northern Canada. *Zoonoses and Public Health* **68**, 601–608 (2021).

53. K. Liu, *et al.*, Effect of climatic factors on the seasonal fluctuation of human brucellosis in Yulin, northern China. *BMC Public Health* **20**, 506 (2020).

54. N. Choubdar, *et al.*, Effect of Meteorological Factors on Hyalomma Species Composition and Their Host Preference, Seasonal Prevalence and Infection Status to Crimean-Congo Haemorrhagic Fever in Iran. *J Arthropod Borne Dis* **13**, 268–283 (2019).

55. A. Sumi, *et al.*, Effect of temperature, relative humidity and rainfall on dengue fever and leptospirosis infections in Manila, the Philippines. *Epidemiol. Infect.* **145**, 78–86 (2017).

56. W. Sun, *et al.*, Effects and interaction of meteorological factors on hemorrhagic fever with renal syndrome incidence in Huludao City, northeastern China, 2007–2018. *PLoS Negl Trop Dis* **15**, e0009217 (2021).

57. Y. Bai, *et al.*, Effects of Climate and Rodent Factors on Hemorrhagic Fever with Renal Syndrome in Chongqing, China, 1997–2008. *PLoS ONE* **10**, e0133218 (2015).

58. Q. Zhao, *et al.*, Effects of climate factors on hemorrhagic fever with renal syndrome in Changchun, 2013 to 2017. *Medicine* **98**, e14640 (2019).

59. A. Jamshidi, A. Haniloo, A. Fazaeli, M. A. Ghatee, Effects of geographical and climatic factors on cystic echinococcosis in south-western Iran. *J. Helminthol.* **94**, e175 (2020).

60. L.-D. Gao, *et al.*, Effects of Humidity Variation on the Hantavirus Infection and Hemorrhagic Fever with Renal Syndrome Occurrence in Subtropical China. *The American Journal of Tropical Medicine and Hygiene* **94**, 420–427 (2016).

61. J. D. Gutierrez, Effects of meteorological factors on human leptospirosis in Colombia. *Int J Biometeorol* **65**, 257–263 (2021).

62. L. P. Yang, *et al.*, Effects of meteorological factors on scrub typhus in a temperate region of China. *Epidemiol. Infect.* **142**, 2217–2226 (2014).

63. J. D. Gutiérrez, *et al.*, Environmental and socioeconomic determinants of leptospirosis incidence in Colombia. *Cad Saude Publica* **35**, e00118417 (2019).

64. E. Hernandez, R. Torres, A. L. Joyce, Environmental and Sociological Factors Associated with the Incidence of West Nile Virus Cases in the Northern San Joaquin Valley of California, 2011-2015. *Vector Borne Zoonotic Dis* **19**, 851–858 (2019).

65. C. Linard, K. Tersago, H. Leirs, E. F. Lambin, Environmental conditions and Puumala virus transmission in Belgium. *Int J Health Geogr* **6**, 55 (2007).

66. F. M. Vescio, *et al.*, Environmental correlates of crimean-congo haemorrhagic fever incidence in Bulgaria. *BMC Public Health* **12**, 1116 (2012).

67. Z. Tolnai, Z. Széll, T. Sréter, Environmental determinants of the spatial distribution of Echinococcus multilocularis in Hungary. *Veterinary Parasitology* **198**, 292–297 (2013).

68. B.-A. Mandja, *et al.*, Environmental Drivers of Monkeypox Transmission in the Democratic Republic of the Congo. *EcoHealth* **19**, 354–364 (2022).

69. S. Zhao, *et al.*, Environmental factors and spatiotemporal distribution of Japanese encephalitis after vaccination campaign in Guizhou Province, China (2004–2016). *BMC Infect Dis* **21**, 1172 (2021).

70. P. L. Bulterys, T. Le, V. M. Quang, K. E. Nelson, J. O. Lloyd-Smith, Environmental Predictors and Incubation Period of AIDS-Associated *Penicillium marneffei* Infection in Ho Chi Minh City, Vietnam. *Clin Infect Dis.* **56**, 1273–1279 (2013).

71. A. Tran, *et al.*, Environmental predictors of West Nile fever risk in Europe. *Int J Health Geogr* **13**, 26 (2014).

72. J.-F. Viel, *et al.*, Environmental risk factors for haemorrhagic fever with renal syndrome in a French new epidemic area. *Epidemiol. Infect.* **139**, 867–874 (2011).

73. M. A. Ghatee, *et al.*, Environmental, climatic and host population risk factors of human cystic echinococcosis in southwest of Iran. *BMC Public Health* **20**, 1611 (2020).

74. Y.-J. Li, X.-L. Li, S. Liang, L.-Q. Fang, W.-C. Cao, Epidemiological features and risk factors associated with the spatial and temporal distribution of human brucellosis in China. *BMC Infect Dis* **13**, 547 (2013).

75. D. Benacer, *et al.*, Epidemiology of human leptospirosis in Malaysia, 2004–2012. *Acta Tropica* **157**, 162–168 (2016).

76. C. Boston, R. Kurup, Estimated Effects of Climate Variables on Transmission of Malaria, Dengue and Leptospirosis within Georgetown, Guyana. *West Indian Med J* (2017). https://doi.org/10.7727/wimj.2017.118.

77. V. Andreo, *et al.*, Estimating Hantavirus Risk in Southern Argentina: A GIS-Based Approach Combining Human Cases and Host Distribution. *Viruses* **6**, 201–222 (2014).

78. F. Shi, *et al.*, Exploring the Dynamics of Hemorrhagic Fever with Renal Syndrome Incidence in East China Through Seasonal Autoregressive Integrated Moving Average Models. *IDR* **Volume 13**, 2465–2475 (2020).

79. C. Smith, C. Skelly, N. Kung, B. Roberts, H. Field, Flying-Fox Species Density - A Spatial Risk Factor for Hendra Virus Infection in Horses in Eastern Australia. *PLoS ONE* **9**, e99965 (2014).

80. H. Bagheri, *et al.*, Forecasting the monthly incidence rate of brucellosis in west of Iran using time series and data mining from 2010 to 2019. *PLoS ONE* **15**, e0232910 (2020).

81. T. Dub, *et al.*, Game Animal Density, Climate, and Tick-Borne Encephalitis in Finland, 2007–2017. *Emerg. Infect. Dis.* **26**, 2899–2906 (2020).

82. P. N. Hamrick, *et al.*, Geographic patterns and environmental factors associated with human yellow fever presence in the Americas. *PLoS neglected tropical diseases* **11**, e0005897 (2017).

83. V. Raharinosy, *et al.*, Geographical distribution and relative risk of Anjozorobe virus (Thailand orthohantavirus) infection in black rats (Rattus rattus) in Madagascar. *Virol J* **15**, 83 (2018).

84. D. W. Redding, *et al.*, Geographical drivers and climate-linked dynamics of Lassa fever in Nigeria. *Nat Commun* **12**, 5759 (2021).

85. K. Tersago, *et al.*, Hantavirus disease (nephropathia epidemica) in Belgium: effects of tree seed production and climate. *Epidemiol. Infect.* **137**, 250–256 (2009).

86. I. Ferro, *et al.*, Hantavirus pulmonary syndrome outbreaks associated with climate variability in Northwestern Argentina, 1997–2017. *PLoS Negl Trop Dis* **14**, e0008786 (2020).

87. R. K. Raghavan, D. G. Goodin, D. Neises, G. A. Anderson, R. R. Ganta, Hierarchical Bayesian Spatio–Temporal Analysis of Climatic and Socio–Economic Determinants of Rocky Mountain Spotted Fever. *PLoS ONE* **11**, e0150180 (2016).

88. D. González-Barrio, *et al.*, Host and Environmental Factors Modulate the Exposure of Free-Ranging and Farmed Red Deer (Cervus elaphus) to Coxiella burnetii. *Applied and Environmental Microbiology* **81**, 6223–6231 (2015).

89. T. T. Hammond, *et al.*, Host biology and environmental variables differentially predict flea abundances for two rodent hosts in a plague-relevant system. *International Journal for Parasitology: Parasites and Wildlife* **9**, 174–183 (2019).

90. S. Zhang, W. Hu, X. Qi, G. Zhuang, How Socio-Environmental Factors Are Associated with Japanese Encephalitis in Shaanxi, China—A Bayesian Spatial Analysis. *IJERPH* **15**, 608 (2018).

91. C. L. Lau, *et al.*, Human Leptospirosis Infection in Fiji: An Eco-epidemiological Approach to Identifying Risk Factors and Environmental Drivers for Transmission. *PLoS Negl Trop Dis* **10**, e0004405 (2016).

92. K. Cucchi, *et al.*, Hydroclimatic drivers of highly seasonal leptospirosis incidence suggest prominent soil reservoir of pathogenic Leptospira spp. in rural western China. *PLoS Negl Trop Dis* **13**, e0007968 (2019).

93. N. I. Stilianakis, *et al.*, Identification of Climatic Factors Affecting the Epidemiology of Human West Nile Virus Infections in Northern Greece. *PLOS ONE* **11**, e0161510 (2016).

94. M. Marcantonio, *et al.*, Identifying the Environmental Conditions Favouring West Nile Virus Outbreaks in Europe. *PLOS ONE* **10**, e0121158 (2015).

95. N. Tokarevich, *et al.*, Impact of air temperature variation on the ixodid ticks habitat and tick-borne encephalitis incidence in the Russian Arctic: the case of the Komi Republic. *International Journal of Circumpolar Health* **76**, 1298882 (2017).

96. F. Ahmadnejad, *et al.*, Impact of climate and environmental factors on west nile virus circulation in Iran. *J. Arthropod-borne Dis.* **10**, 315 (2016).

97. J. Xiang, *et al.*, Impact of meteorological factors on hemorrhagic fever with renal syndrome in 19 cities in China, 2005–2014. *Science of The Total Environment* **636**, 1249–1256 (2018).

98. C. A. Parmenter, R. R. Parmenter, P. Ettestad, K. L. Gage, E. P. Yadav, Incidence of plague associated with increased winter-spring precipitation in New Mexico. *The American Journal of Tropical Medicine and Hygiene* **61**, 814–821 (1999).

99. J. L. Duarte, L. L. Giatti, Incidência da leptospirose em uma capital da Amazônia Ocidental brasileira e sua relação com a variabilidade climática e ambiental, entre os anos de 2008 e 2013*. *Epidemiologia e Serviços de Saúde* **28** (2019).

100. A. Major, A. Schweighauser, T. Francey, Increasing Incidence of Canine Leptospirosis in Switzerland. *IJERPH* **11**, 7242–7260 (2014).

101. J. Wu, *et al.*, Increasing incidence of hemorrhagic fever with renal syndrome could be associated with livestock husbandry in Changchun, Northeastern China. *BMC Infect Dis* **14**, 301 (2014).

102. J. E. Soverow, G. A. Wellenius, D. N. Fisman, M. A. Mittleman, Infectious Disease in a Warming World: How Weather Influenced West Nile Virus in the United States (2001–2005). *Environmental Health Perspectives* **117**, 1049–1052 (2009).

103. H. Zheng, *et al.*, Influence and prediction of meteorological factors on brucellosis in a northwest region of China. *Environ Sci Pollut Res* **30**, 9962–9973 (2022).

104. T. Batchelor, T. Stephenson, P. Brown, D. Amarakoon, M. Taylor, Influence of climate variability on human leptospirosis cases in Jamaica. *Clim. Res.* **55**, 79–90 (2012).

105. S. Paz, I. Albersheim, Influence of Warming Tendency on Culex pipiens Population Abundance and on the Probability of West Nile Fever Outbreaks (Israeli Case Study: 2001–2005). *EcoHealth* **5**, 40–48 (2008).

106. W. J. Landesman, B. F. Allan, R. B. Langerhans, T. M. Knight, J. M. Chase, Inter-Annual Associations Between Precipitation and Human Incidence of West Nile Virus in the United States. *Vector-Borne and Zoonotic Diseases* **7**, 337–343 (2007).

107. H. S. Young, *et al.*, Interacting effects of land use and climate on rodent-borne pathogens in central Kenya. *Phil. Trans. R. Soc. B* **372**, 20160116 (2017).

108. G. Titcomb, *et al.*, Interacting effects of wildlife loss and climate on ticks and tick-borne disease. *Proc. R. Soc. B.* **284**, 20170475 (2017).

109. L. Cao, *et al.*, Interactions and marginal effects of meteorological factors on haemorrhagic fever with renal syndrome in different climate zones: Evidence from 254 cities of China. *Science of The Total Environment* **721**, 137564 (2020).

110. H. Tian, *et al.*, Interannual cycles of Hantaan virus outbreaks at the human–animal interface in Central China are controlled by temperature and rainfall. *Proc. Natl. Acad. Sci. U.S.A.* **114**, 8041–8046 (2017).

111. Y. Li, *et al.*, Intrinsic and extrinsic drivers of transmission dynamics of hemorrhagic fever with renal syndrome caused by Seoul hantavirus. *PLoS Negl Trop Dis* **13**, e0007757 (2019).

112. P. Guan, *et al.*, Investigating the effects of climatic variables and reservoir on the incidence of hemorrhagic fever with renal syndrome in Huludao City, China: a 17-year data analysis based on structure equation model. *BMC Infect Dis* **9**, 109 (2009).

113. H. Xiao, *et al.*, Investigating the Effects of Food Available and Climatic Variables on the Animal Host Density of Hemorrhagic Fever with Renal Syndrome in Changsha, China. *PLoS ONE* **8**, e61536 (2013).

114. R. Miramontes, W. E. Lafferty, B. K. Lind, M. W. Oberle, Is Agricultural Activity Linked to the Incidence of Human West Nile Virus? *American Journal of Preventive Medicine* **30**, 160–163 (2006).

115. H. Singh, N. Singh, R. K. Mall, Japanese Encephalitis and Associated Environmental Risk Factors in Eastern Uttar Pradesh: A time series analysis from 2001 to 2016. *Acta Tropica* **212**, 105701 (2020).

116. X. Zhao, *et al.*, Japanese Encephalitis Risk and Contextual Risk Factors in Southwest China: A Bayesian Hierarchical Spatial and Spatiotemporal Analysis. *IJERPH* **11**, 4201–4217 (2014).

117. C. B. Zeimes, *et al.*, Landscape and Regional Environmental Analysis of the Spatial Distribution of Hantavirus Human Cases in Europe. *Front. Public Health* **3** (2015).

118. J. P. Langlois, L. Fahrig, G. Merriam, H. Artsob, Landscape structure influences continental distribution of hantavirus in deer mice. *Landscape Ecology* **16**, 255–266 (2001).

119. J. P. DeGroote, R. Sugumaran, M. Ecker, Landscape, demographic and climatic associations with human west nile virus occurrence regionally in 2012 in the United States of america. *Geospat. Health* **9**, 153 (2014).

120. J. P. DeGroote, R. Sugumaran, S. M. Brend, B. J. Tucker, L. C. Bartholomay, Landscape, demographic, entomological, and climatic associations with human disease incidence of west nile virus in the state of iowa, USA. *Int. J. Health Geographics* **7**, 19 (2008).

121. P. R. Prist, *et al.*, Landscape, Environmental and Social Predictors of Hantavirus Risk in São Paulo, Brazil. *PLoS ONE* **11**, e0163459 (2016).

122. M. F. Mohd Radi, *et al.*, Leptospirosis Outbreak After the 2014 Major Flooding Event in Kelantan, Malaysia: A Spatial-Temporal Analysis. *The American Journal of Tropical Medicine and Hygiene* **98**, 1281–1295 (2018).

123. Y. Ma, *et al.*, Linking climate and infectious disease trends in the Northern/Arctic Region. *Sci Rep* **11**, 20678 (2021).

124. W.-J. Chen, *et al.*, Mapping the Distribution of Anthrax in Mainland China, 2005–2013. *PLoS Negl Trop Dis* **10**, e0004637 (2016).

125. R. Bargaoui, S. Lecollinet, R. Lancelot, Mapping the Serological Prevalence Rate of West Nile fever in Equids, Tunisia. *Transbound Emerg Dis* **62**, 55–66 (2015).

126. M. B. Hahn, *et al.*, Meteorological Conditions Associated with Increased Incidence of West Nile Virus Disease in the United States, 2004–2012. *Am J Trop Med Hyg* **92**, 1013–1022 (2015).

127. R. S. Nasci, *et al.*, Meteorological Conditions Associated with Increased Incidence of West Nile Virus Disease in the United States, 2004–2012. *The American Journal of Tropical Medicine and Hygiene* **92**, 1013–1022 (2015).

128. F. Jiang, *et al.*, Meteorological factors affect the epidemiology of hemorrhagic fever with renal syndrome via altering the breeding and hantavirus-carrying states of rodents and mites: a 9 years' longitudinal study. *Emerging Microbes & Infections* **6**, 1–9 (2017).

129. J. Seto, *et al.*, Meteorological factors affecting scrub typhus occurrence: a retrospective study of Yamagata Prefecture, Japan, 1984–2014. *Epidemiol. Infect.* **145**, 462–470 (2017).

130. Y. Wei, *et al.*, Meteorological factors and risk of hemorrhagic fever with renal syndrome in Guangzhou, southern China, 2006–2015. *PLoS Negl Trop Dis* **12**, e0006604 (2018).

131. T. Li, Z. Yang, Z. Dong, M. Wang, Meteorological factors and risk of scrub typhus in Guangzhou, southern China, 2006–2012. *BMC Infect Dis* **14**, 139 (2014).

132. H. Lin, Z. Zhang, L. Lu, X. Li, Q. Liu, Meteorological factors are associated with hemorrhagic fever with renal syndrome in Jiaonan County, China, 2006–2011. *Int J Biometeorol* **58**, 1031–1037 (2014).

133. A. Mavrakis, C. Papavasileiou, D. Alexakis, E. C. Papakitsos, L. Salvati, Meteorological patterns and the evolution of West Nile virus in an environmentally stressed Mediterranean area. *Environ Monit Assess* **193**, 227 (2021).

134. V. Andreo, G. Glass, T. Shields, C. Provensal, J. Polop, Modeling Potential Distribution of Oligoryzomys longicaudatus, the Andes Virus (Genus: Hantavirus) Reservoir, in Argentina. *EcoHealth* **8**, 332–348 (2011).

135. S. Chadsuthi, C. Modchang, Y. Lenbury, S. Iamsirithaworn, W. Triampo, Modeling seasonal leptospirosis transmission and its association with rainfall and temperature in Thailand using time–series and ARIMAX analyses. *Asian Pacific Journal of Tropical Medicine* **5**, 539–546 (2012).

136. E. O. Nsoesie, S. R. Mekaru, N. Ramakrishnan, M. V. Marathe, J. S. Brownstein, Modeling to Predict Cases of Hantavirus Pulmonary Syndrome in Chile. *PLoS Negl Trop Dis* **8**, e2779 (2014).

137. G. Marini, *et al.*, Modelling the West Nile virus force of infection in the European human population. *One Health* **15**, 100462 (2022).

138. J. Habus, *et al.*, New trends in human and animal leptospirosis in Croatia, 2009–2014. *Acta Tropica* **168**, 1–8 (2017).

139. B. Bhopdhornangkul, *et al.*, Non-linear effect of different humidity types on scrub typhus occurrence in endemic provinces, Thailand. *Heliyon* **7**, e06095 (2021).

140. Z. Liu, *et al.*, Nonlinear and Threshold Effect of Meteorological Factors on Japanese Encephalitis Transmission in Southwestern China. *The American Journal of Tropical Medicine and Hygiene* **103**, 2442–2449 (2020).

141. S. Paz, *et al.*, Permissive Summer Temperatures of the 2010 European West Nile Fever Upsurge. *PLOS ONE* **8**, e56398 (2013).

142. K. Tersago, *et al.*, Population, Environmental, and Community Effects on Local Bank Vole (*Myodes glareolus*) Puumala Virus Infection in an Area with Low Human Incidence. *Vector-Borne and Zoonotic Diseases* **8**, 235–244 (2008).

143. H. Faramarzi, M. Nasiri, M. Khosravi, A. Keshavarzi, A. R. Rezaei Ardakani, Potential Effects of Climatic Parameters on Human Brucellosis in Fars Province, Iran, during 2009-2015. *Iranian Journal of Medical Sciences* **44** (2019).

144. K. Hampson, *et al.*, Predictability of anthrax infection in the Serengeti, Tanzania. *Journal of Applied Ecology* **48**, 1333–1344 (2011).

145. M. Mohammadian-Khoshnoud, M. Sadeghifar, Z. Cheraghi, Z. Hosseinkhani, Predicting the incidence of brucellosis in Western Iran using Markov switching model. *BMC Res Notes* **14**, 79 (2021).

146. P. M. Munyua, *et al.*, Predictive Factors and Risk Mapping for Rift Valley Fever Epidemics in Kenya. *PLoS ONE* **11**, e0144570 (2016).

147. R. M. Srinivasa, M. U. Suryanarayana, N. Arunachalam, Prevalence of japanese encephalitis and its modulation by weather variables. *J. Public Health Epidemiol.* **6**, 52–59 (2014).

148. S. Nusinovici, J. Frössling, S. Widgren, F. Beaudeau, A. Lindberg, Q fever infection in dairy cattle herds: increased risk with high wind speed and low precipitation. *Epidemiology & Infection* **143**, 3316–3326 (2015).

149. M. Cunha, *et al.*, Rainfall and other meteorological factors as drivers of urban transmission of leptospirosis. *PLoS Negl Trop Dis* **16**, e0007507 (2022).

150. A. Rizzoli, *et al.*, Recent increase in prevalence of antibodies to Dobrava-Belgrade virus (DOBV) in yellow-necked mice in northern Italy. *Epidemiol. Infect.* **143**, 2241–2244 (2015).

151. J. I. Rosser, K. Nielsen-Saines, E. Saad, T. Fuller, Reemergence of yellow fever virus in southeastern Brazil, 2017–2018: What sparked the spread? *PLOS Neglected Tropical Diseases* **16**, e0010133 (2022).

152. Y. Bai, *et al.*, Regional Impact of Climate on Japanese Encephalitis in Areas Located near the Three Gorges Dam. *PLoS ONE* **9**, e84326 (2014).

153. M. C. Wimberly, A. Lamsal, P. Giacomo, T.-W. Chuang, Regional Variation of Climatic Influences on West Nile Virus Outbreaks in the United States. *Am J Trop Med Hyg* **91**, 677–684 (2014).

154. J. Clement, *et al.*, Relating increasing hantavirus incidences to the changing climate: the mast connection. *Int J Health Geogr* **8**, 1 (2009).

155. L. Cao, *et al.*, Relationship of meteorological factors and human brucellosis in Hebei province, China. *Science of The Total Environment* **703**, 135491 (2020).

156. A. C. Schwarz, U. Ranft, I. Piechotowski, J. E. Childs, S. O. Brockmann, Risk Factors for Human Infection with Puumala Virus, Southwestern Germany. *Emerg. Infect. Dis.* **15**, 1032–1039 (2009).

157. M. Maroli, M. V. Vadell, P. Padula, I. E. G. Villafañe, Rodent Abundance and Hantavirus Infection in Protected Area, East-Central Argentina. *Emerg. Infect. Dis.* **24**, 131–134 (2018).

158. J. Kwak, *et al.*, Scrub Typhus Incidence Modeling with Meteorological Factors in South Korea. *IJERPH* **12**, 7254–7273 (2015).

159. P.-J. Tsai, H.-C. Yeh, Scrub typhus islands in the Taiwan area and the association between scrub typhus disease and forest land use and farmer population density: geographically weighted regression. *BMC Infect Dis* **13**, 191 (2013).

160. A. Hamlet, K. A. Gaythorpe, T. Garske, N. M. Ferguson, Seasonal and inter-annual drivers of yellow fever transmission in South America. *PLoS neglected tropical diseases* **15**, e0008974 (2021).

161. C.-L. Lin, H.-L. Chang, C.-Y. Lin, K.-T. Chen, Seasonal Patterns of Japanese Encephalitis and Associated Meteorological Factors in Taiwan. *IJERPH* **14**, 1317 (2017).

162. A. C. Keyel, *et al.*, Seasonal temperatures and hydrological conditions improve the prediction of West Nile virus infection rates in Culex mosquitoes and human case counts in New York and Connecticut. *PLoS One* **14**, e0217854 (2019).

163. A. Hamlet, *et al.*, Seasonality of agricultural exposure as an important predictor of seasonal yellow fever spillover in Brazil. *Nature communications* **12**, 3647 (2021).

164. A. Desvars, *et al.*, Seasonality of Human Leptospirosis in Reunion Island (Indian Ocean) and Its Association with Meteorological Data. *PLoS ONE* **6**, e20377 (2011).

165. V. Marin, J. Mellado, J. García, L. Gaytán, M. Mellado, Seroprevalence and Risk Factors for Brucellosis in Free-Range Goats. *Israel Journal of Veterinary Medicine* **71**, 14–20 (2016).

166. M. F. Vescio, *et al.*, Socio-demographic and climatic factors as correlates of Mediterranean spotted fever (MSF) in northern Sardinia. *Am J Trop Med Hyg* **78**, 318–320 (2008).

167. M. Ahmadkhani, A. A. Alesheikh, Department of Geo-spatial Information System (GIS), K.N.Toosi University of Technology, Tehran, Iran, Space-time analysis of human brucellosis considering environmental factors in Iran. *APJTD* **7**, 257–265 (2017).

168. C.-C. Kuo, J.-L. Huang, C.-Y. Ko, P.-F. Lee, H.-C. Wang, Spatial analysis of scrub typhus infection and its association with environmental and socioeconomic factors in Taiwan. *Acta Tropica* **120**, 52–58 (2011).

169. L. A. Reperant, N. S. Fučkar, A. D. M. E. Osterhaus, A. P. Dobson, T. Kuiken, Spatial and Temporal Association of Outbreaks of H5N1 Influenza Virus Infection in Wild Birds with the 0°C Isotherm. *PLoS Pathog* **6**, e1000854 (2010).

170. P. M. Muñoz, *et al.*, Spatial distribution and risk factors of Brucellosis in Iberian wild ungulates. *BMC Infect Dis* **10**, 46 (2010).

171. Spatial distribution and the impact of geographical factors on brucellosis in Chaharmahal and Bakhtiari Province, Iran. *Int J Epidemiol Res* **3**, 98–105 (2016).

172. P. W. Dhewantara, *et al.*, Spatial distribution of leptospirosis incidence in the Upper Yangtze and Pearl River Basin, China: Tools to support intervention and elimination. *Science of The Total Environment* **725**, 138251 (2020).

173. C. Peng, Y.-J. Li, D.-S. Huang, P. Guan, Spatial-temporal distribution of human brucellosis in mainland China from 2004 to 2017 and an analysis of social and environmental factors. *Environ Health Prev Med* **25**, 1 (2020).

174. D. W. Redding, S. Tiedt, G. Lo Iacono, B. Bett, K. E. Jones, Spatial, seasonal and climatic predictive models of Rift Valley fever disease across Africa. *Phil. Trans. R. Soc. B* **372**, 20160165 (2017).

175. K. Mogano, *et al.*, Spatio-temporal epidemiology of animal and human rabies in northern South Africa between 1998 and 2017. *PLoS Negl Trop Dis* **16**, e0010464 (2022).

176. L. Ge, *et al.*, Spatio-Temporal Pattern and Influencing Factors of Hemorrhagic Fever with Renal Syndrome (HFRS) in Hubei Province (China) between 2005 and 2014. *PLoS ONE* **11**, e0167836 (2016).

177. S. Radojicic, *et al.*, Spatiotemporal Analysis of West Nile Virus Epidemic in South Banat District, Serbia, 2017-2019. *Animals (Basel)* **11** (2021).

178. D. Liang, *et al.*, Spatiotemporal distribution of human brucellosis in Inner Mongolia, China, in 2010–2015, and influencing factors. *Sci Rep* **11**, 24213 (2021).

179. Y.-C. Wu, *et al.*, Spatiotemporal Dynamics of Scrub Typhus Transmission in Mainland China, 2006-2014. *PLoS Negl Trop Dis* **10**, e0004875 (2016).

180. Z. Yang, *et al.*, Spatiotemporal expansion of human brucellosis in Shaanxi Province, Northwestern China and model for risk prediction. *PeerJ* **8**, e10113 (2020).

181. S. Li, *et al.*, Spatiotemporal Heterogeneity Analysis of Hemorrhagic Fever with Renal Syndrome in China Using Geographically Weighted Regression Models. *IJERPH* **11**, 12129–12147 (2014).

182. L.-Q. Fang, *et al.*, Spatiotemporal Trends and Climatic Factors of Hemorrhagic Fever with Renal Syndrome Epidemic in Shandong Province, China. *PLoS Negl Trop Dis* **4**, e789 (2010).

183. V. Naveenkumar, *et al.*, Temporal pattern and risk factors for occurrence of Canine Rabies in Chennai. *Comparative Immunology, Microbiology and Infectious Diseases* **90–91**, 101903 (2022).

184. X. Liu, B. Jiang, W. Gu, Q. Liu, Temporal trend and climate factors of hemorrhagic fever with renal syndrome epidemic in Shenyang City, China. *BMC Infect Dis* **11**, 331 (2011).

185. A. E. P. Silva, M. D. R. D. D. O. Latorre, F. Chiaravalloti Neto, G. M. D. S. Conceição, Tendência temporal da leptospirose e sua associação com variáveis climáticas e ambientais em Santa Catarina, Brasil. *Ciênc. saúde coletiva* **27**, 849–860 (2022).

186. S. Boqvist, L. Eliasson-Selling, K. Bergström, U. Magnusson, The association between rainfall and seropositivity to Leptospira in outdoor reared pigs. *The Veterinary Journal* **193**, 135–139 (2012).

187. C. D. McKee, *et al.*, The Ecology of Nipah Virus in Bangladesh: A Nexus of Land-Use Change and Opportunistic Feeding Behavior in Bats. *Viruses* **13**, 169 (2021).

188. S. Nili, N. Khanjani, Y. Jahani, B. Bakhtiari, The effect of climate variables on the incidence of Crimean Congo Hemorrhagic Fever (CCHF) in Zahedan, Iran. *BMC Public Health* **20**, 1893 (2020).

189. I. Kurane, K. Shibasaki, A. Kotaki, Y. Hijioka, T. Takasaki, The Effect of Precipitation on the Transmission of Japanese Encephalitis (JE) Virus in Nature: A Complex Effect on Antibody-Positive Rate to JE Virus in Sentinel Pigs. *IJERPH* **10**, 1831–1844 (2013).

190. H. M. Munang'andu, *et al.*, The effect of seasonal variation on anthrax epidemiology in the upper Zambezi floodplain of western Zambia. *J Vet Sci* **13**, 293 (2012).

191. T. Wangrangsimakul, *et al.*, The estimated burden of scrub typhus in Thailand from national surveillance data (2003-2018). *PLoS Negl Trop Dis* **14**, e0008233 (2020).

192. N. K. Tokarevich, *et al.*, The impact of climate change on the expansion of *Ixodes persulcatus* habitat and the incidence of tick-borne encephalitis in the north of European Russia. *Global Health Action* **4**, 8448 (2011).

193. S. M. Hsu, A. M. F. Yen, T. H. H. Chen, The impact of climate on Japanese encephalitis. *Epidemiol. Infect.* **136**, 980–987 (2008).

194. M. S. Z. S. Coelho, E. Massad, The impact of climate on Leptospirosis in São Paulo, Brazil. *Int J Biometeorol* **56**, 233–241 (2012).

195. E. Britton, S. Hales, K. Venugopal, M. G. Baker, The impact of climate variability and change on cryptosporidiosis and giardiasis rates in New Zealand. *Journal of Water and Health* **8**, 561–571 (2010).

196. J. Yin, X. Wu, C. Li, J. Han, H. Xiang, The impact of environmental factors on human echinococcosis epidemics: spatial modelling and risk prediction. *Parasites Vectors* **15**, 47 (2022).

197. J. Yin, X. Wu, J. Han, P. R. Torgerson, The impact of natural environment on human alveolar echinococcosis: A township-level modeling study in Qinghai-Tibet Plateau. *Science of The Total Environment* **856**, 159085 (2023).

198. J. He, *et al.*, The Impacts of Climatic Factors and Vegetation on Hemorrhagic Fever with Renal Syndrome Transmission in China: A Study of 109 Counties. *IJERPH* **16**, 3434 (2019).

199. A. E. Platonov, *et al.*, The Incidence of West Nile Disease in Russia in Relation to Climatic and Environmental Factors. *International Journal of Environmental Research and Public Health* **11**, 1211–1232 (2014).

200. Y. P. Joshi, E.-H. Kim, H.-K. Cheong, The influence of climatic factors on the development of hemorrhagic fever with renal syndrome and leptospirosis during the peak season in Korea: an ecologic study. *BMC Infect Dis* **17**, 406 (2017).

201. M. G. Walsh, M. Haseeb, The landscape configuration of zoonotic transmission of Ebola virus disease in West and Central Africa: interaction between population density and vegetation cover. *PeerJ* **3**, e735 (2015).

202. M. J. Watts, V. Sarto i Monteys, P. G. Mortyn, P. Kotsila, The rise of West Nile Virus in Southern and Southeastern Europe: A spatial–temporal analysis investigating the combined effects of climate, land use and economic changes. *One Health* **13**, 100315 (2021).

203. M. G. Walsh, The Role of Hydrogeography and Climate in the Landscape Epidemiology of West Nile Virus in New York State from 2000 to 2010. *PLOS ONE* **7**, e30620 (2012).

204. J. I. Nchom, A. S. Abubakar, F. O. Arimoro, B. Y. Mohammed, The Role of Weather in the Spread of Lassa Fever in Parts of Northern Nigeria. *IJTDH* 33–40 (2021). https://doi.org/10.9734/ijtdh/2021/v42i2330562.

205. L. J. Fairhead, *et al.*, The seasonality of infections in tropical Far North Queensland, Australia: A 21-year retrospective evaluation of the seasonal patterns of six endemic pathogens. *PLOS Glob Public Health* **2**, e0000506 (2022).

206. D. E. Impoinvil, *et al.*, The Spatial Heterogeneity between Japanese Encephalitis Incidence Distribution and Environmental Variables in Nepal. *PLoS ONE* **6**, e22192 (2011).

207. S. Paz, The west nile virus outbreak in Israel (2000) from a new perspective: The regional impact of climate change. *International Journal of Environmental Health Research* **16**, 1–13 (2006).

208. G. Carpi, F. Cagnacci, M. Neteler, A. Rizzoli, Tick infestation on roe deer in relation to geographic and remotely sensed climatic variables in a tick-borne encephalitis endemic area. *Epidemiol. Infect.* **136**, 1416–1424 (2008).

209. E. Lindgren, R. Gustafson, Tick-borne encephalitis in Sweden and climate change. *The Lancet* **358**, 16–18 (2001).

210. H. Lin, *et al.*, Time series analysis of Japanese encephalitis and weather in Linyi City, China. *Int J Public Health* **57**, 289–296 (2012).

211. J. Warnasekara, S. Agampodi, R. Abeynayake R., Time series models for prediction of leptospirosis in different climate zones in Sri Lanka. *PLoS ONE* **16**, e0248032 (2021).

212. E. Afonso, P. Thulliez, E. Gilot-Fromont, Transmission of Toxoplasma gondii in an urban population of domestic cats (Felis catus). *International Journal for Parasitology* **36**, 1373–1382 (2006).

213. S. Colombe, *et al.*, Trends and correlates of cystic echinococcosis in Chile: 2001–2012. *PLoS Negl Trop Dis* **11**, e0005911 (2017).

214. K. H. Smith, *et al.*, Using Climate to Explain and Predict West Nile Virus Risk in Nebraska. *GeoHealth* **4**, e2020GH000244 (2020).

215. W. Hu, S. Tong, K. Mengersen, D. Connell, Weather Variability and the Incidence of Cryptosporidiosis: Comparison of Time Series Poisson Regression and SARIMA Models. *Annals of Epidemiology* **17**, 679–688 (2007).

216. P. Bi, Y. Zhang, K. A. Parton, Weather variables and Japanese encephalitis in the metropolitan area of Jinan city, China. *Journal of Infection* **55**, 551–556 (2007).

217. B. V. Giordano, S. Kaur, F. F. Hunter, West Nile virus in Ontario, Canada: A twelve-year analysis of human case prevalence, mosquito surveillance, and climate data. *PLOS ONE* **12**, e0183568 (2017).

218. D. W. Crowder, *et al.*, West Nile Virus Prevalence across Landscapes Is Mediated by Local Effects of Agriculture on Vector and Host Communities. *PLoS ONE* **8**, e55006 (2013).
